# Cherbonolides M and N from a Formosan Soft Coral *Sarcophyton* *cherbonnieri*

**DOI:** 10.3390/md19050260

**Published:** 2021-05-01

**Authors:** Chia-Chi Peng, Tzu-Yin Huang, Chiung-Yao Huang, Tsong-Long Hwang, Jyh-Horng Sheu

**Affiliations:** 1 Department of Marine Biotechnology and Resources, National Sun Yat-Sen University, Kaohsiung 804, Taiwan; Chia-Chi.Peng@hki-jena.de (C.-C.P.); huangcy@mail.nsysu.edu.tw (C.-Y.H.); 2 Doctoral Degree Program in Marine Biotechnology, National Sun Yat-Sen University, Kaohsiung 804, Taiwan; HuangTY@g-mail.nsysu.edu.tw; 3 Graduate Institute of Natural Products, College of Medicine, Chang Gung University, Taoyuan 333, Taiwan; htl@mail.cgu.edu.tw; 4 Research Center for Industry of Human Ecology and Graduate Institute of Health Industry Technology, Chang Gung University of Science and Technology, Taoyuan 333, Taiwan; 5 Department of Anesthesiology, Chang Gung Memorial Hospital, Taoyuan 333, Taiwan; 6 Graduate Institute of Natural Products, Kaohsiung Medical University, Kaohsiung 807, Taiwan; 7 Department of Medical Research, China Medical University Hospital, China Medical University, Taichung 404, Taiwan

**Keywords:** isosarcophine derivatives, *Sarcophyton cherbonnieri*, tetrahydrooxepane, cytotoxicity, anti-inflammatory activity

## Abstract

Two new isosarcophine derivatives, cherbonolides M (**1**) and N (**2**), were further isolated from a Formosan soft coral *Sarcophyton cherbonnieri*. The planar structure and relative configuration of both compounds were established by the detailed analysis of the IR, MS, and 1D and 2D NMR data. Further, the absolute configuration of both compounds was determined by the comparison of CD spectra with that of isosarcophine (**3**). Notably, cherbonolide N (**2**) possesses the unique cembranoidal scaffold of tetrahydrooxepane with the 12,17-ether linkage fusing with a *γ*-lactone. In addition, the assay for cytotoxicity of both new compounds revealed that they showed to be noncytotoxic toward the proliferation of A549, DLD-1, and HuCCT-1 cell lines. Moreover, the anti-inflammatory activities of both metabolites were carried out by measuring the *N*-formyl-methionyl-leucyl-phenylalanine/cytochalasin B (fMLF/CB)-induced generation of superoxide anion and elastase release in the primary human neutrophils. Cherbonolide N (**2**) was found to reduce the generation of superoxide anion (20.6 ± 6.8%) and the elastase release (30.1 ± 3.3%) in the fMLF/CB-induced human neutrophils at a concentration of 30 μM.

## 1. Introduction

The chemical constituents for soft corals of the genera *Sarcophyton* have been well studied. According to the statistics, more than 500 marine natural products have been isolated from this genus during the past two decades. Among these metabolites, over 300 diterpenoids with the 14 membered cembranoidal skeleton were discovered [1]. Therefore, the *Sarcophyton* genus has been frequently considered to be an important source of the 14 membered ring diterpenoidal skeleton [2,3,4]. These secondary metabolites help the organisms to defend themselves against the predators and adapt to the environment stress [5]. Furthermore, some of the isolates have been demonstrated to possess various biological activities, such as cytotoxic [6,7,8,9,10,11,12], anti-inflammatory [6,7,13,14,15,16,17,18], neuroprotective [19], antibacterial [19], and antiviral activities [12,20]. The diversified structures and various biological applications of marine natural products thus prompt us to further explore the secondary metabolites from organisms of the *Sarcophyton* genus. 

Sarcophine, the dihydrofuran-containing cembranoidal diterpene, is one of the major metabolites in the soft corals of *Sarcophyton* genera [21]. Bernstein et al. discovered this metabolite from the soft coral *Sarcophyton glaucum* in 1974 [22]. Subsequently, the absolute configuration of sarcophine was determined by Kashman et al. in 1977 [23]. Furthermore, Frincke et al. indicated that sarcophine was converted from the other 14 membered diterpene, sarcophytoxide, by auto-oxidation in 1980 [24]. Isosarcophine, an isomer of sarcophine, was proven to be converted from isosarcophytoxide via auto-oxidation and isolated from the soft coral *Sinularia mayi* by Kusumi et al. in 1990 [25]. It was found that isosarcophine showed significant cytotoxicities toward some cancer cell lines [25,26]. 

**Figure 1:**
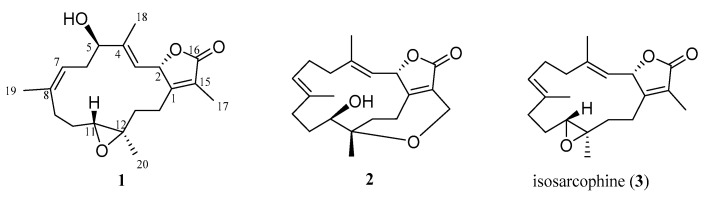
Marine natural products **1**–**3** isolated from the soft coral *S. cherbonnieri.*

Our previous investigation of soft coral *Sarcophyton cherbonnieri* had contributed to the isolation of 13 new cembranoids derived from the isosarcophine [13,14]. In the present study, the continuous chemical investigation of *S. cherbonnieri* resulted in the discovery of two new isosarcophine derivatives, cherbonolides M (**1**) and N (**2**) as shown in Figure 1. The planar structures and relative configurations of both compounds were elucidated by analyzing the infrared (IR), MS, and 1D and 2D NMR data. Furthermore, to determine the absolute configurations, the circular dichroism (CD) spectra of both new compounds were measured and compared with those of isosarcophine (**3**). Moreover, in order to discover bioactive natural products for the development of drug leads, the cytotoxicity against human lung adenocarcinoma (A549), human colorectal adenocarcinoma (DLD-1), and human intrahepatic cholangiocarcinoma (HuCCT-1) was examined. In addition, the anti-inflammatory activity of both isolates was investigated by measuring the *N*-formyl-methionyl-leucyl-phenylalanine/cytochalasin B (fMLF/CB)-induced generation of superoxide anion and elastase release in the primary human neutrophils. 

## 2. Results and Discussion

The chemical structures of metabolites **1** and **2** were elucidated by analyzing the MS, IR, CD, and 1D and 2D NMR data (Supplementary Materials Figures S1–S32). Additionally, the ^13^C and ^1^H chemical shifts of **1** and **2** are listed in Table 1.

Compound **1,** cherbonolide M, was isolated as a colorless oil. The molecular formula of **1** was C_20_H_28_O_4_ deduced from the pseudomolecular ion peak at *m/z* 355.1882 ${\left[M+\mathrm{Na}\right]}^{+}$ (calculated 355.1880, C_20_H_28_O_4_Na) in the high-resolution electrospray ionization mass spectrometry (HR-ESI-MS). Its IR spectrum showed the absorptions at 3445 and 1749 cm^–1^, indicating the presence of hydroxy and ester groups. The ^13^C and distortionless enhancement by polarization transfer (DEPT) spectra displayed 20 carbon signals, including four methyls, five methylenes, five methines, and six quaternary carbons. ^1^H and ^13^C NMR spectra showed the signals of an *α*-methyl-*α*, *β*-unsaturated-*γ*-lactone (*δ*_H_ 5.64, d, *J* = 10.4 Hz; 1.78, s; *δ*_C_ 174.6, C; 162.4, C; 123.5, C; 78.4, CH; 8.6, CH_3_), two trisubstituted double bonds (*δ*_H_ 4.98, d, *J* = 10.4 Hz; *δ*_C_ 123.4, CH; 147.5, C; *δ*_H_ 4.98, d, *J* = 10.8 Hz; *δ*_C_ 122.5, CH; 134.9, C), an oxygen-bearing methine (*δ*_H_ 4.21, dd, *J* = 10.8, 5.2 Hz; *δ*_C_ 77.5, CH), and an epoxy groups (*δ*_H_ 2.45, d, *J* = 10.8 Hz; *δ*_C_ 62.0, CH and 61.1, C).

**Figure 2:**
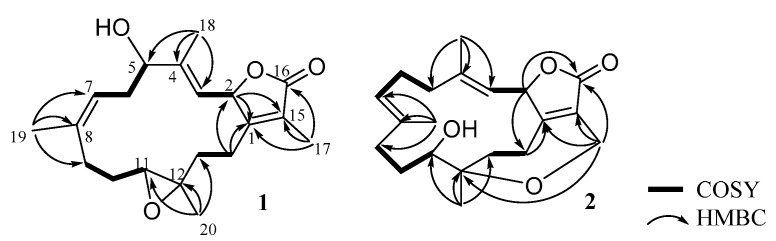
COSY and selective HMBC correlations of **1** and **2**.

The planar structure of **1** was established according to the analysis of 2D NMR spectra as shown in Figure 2. The correlation spectroscopy (COSY) spectrum showed four partial moieties from the correlations of H-2 to H-3, H-5 via H_2_-6 to H-7, H_2_-9 via H_2_-10 to H-11, and H_2_-13 to H_2_-14. These partial structures were assembled by heteronuclear multiple bond correlation (HMBC) correlations from H_3_-17 to C-1, C-15, C-16; H_3_-18 to C-3, C-4, C-5; H_3_-19 to C-7, C-8, C-9; H_3_-20 to C-11, C-12, C-13; and H_2_-14 to C-1 and C-2. Furthermore, the NMR spectroscopic data of **1** were compared with those of the previous metabolite cherbonolide H [13] for structural elucidation. It was shown that compound **1** should possess a hydroxy group at C-5 and a 7,8-trisubstituted double bond from this NMR data comparison. According to the above evidence, the gross structure of **1** was elucidated.

**Figure 3:**
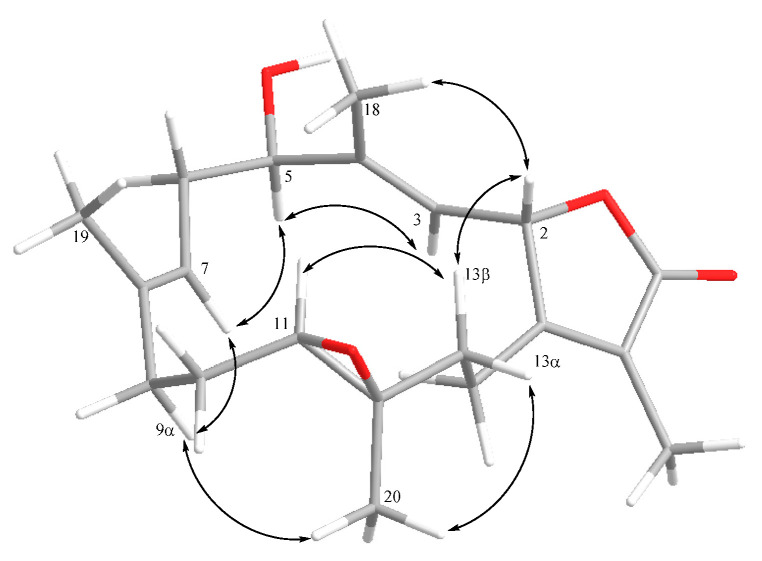
Selective NOE correlations of **1**.

**Figure 4:**
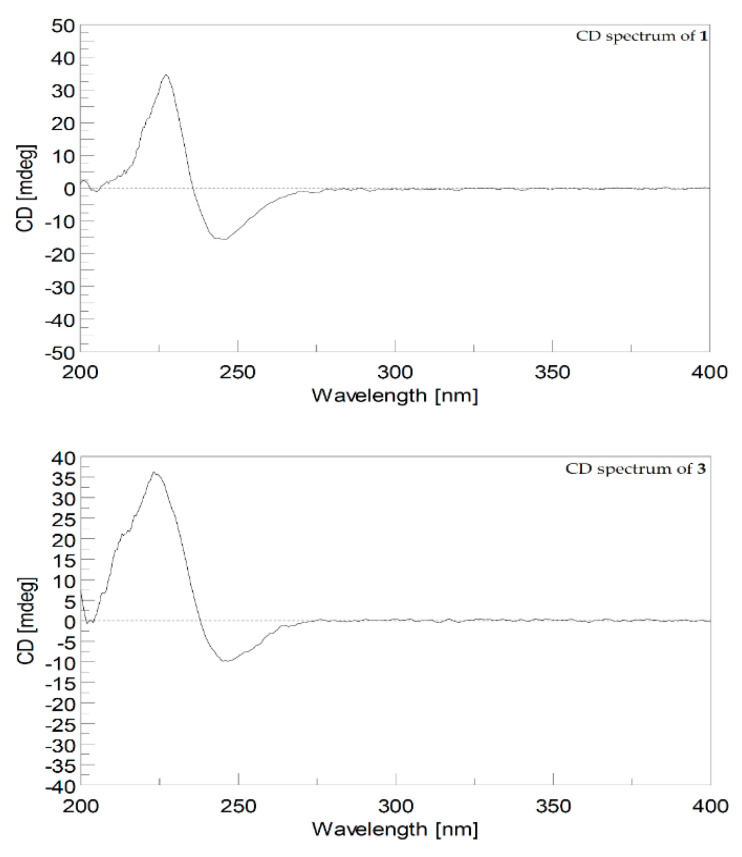
CD spectrum (1.2 × 10^–4^ M, MeOH) of **1** and CD spectrum (1.6 × 10^–4^ M, MeOH) of **3**.

The relative configuration of **1** was established by analyzing the nuclear Overhauser effect spectroscopy (NOESY) spectrum, as shown in Figure 3. Assuming the *β*-orientation of H-2 (*δ*_H_ 5.64, d, *J* = 10.4 Hz), it was found the NOE correlations of H-2 with H-13*β* (*δ*_H_ 1.04, t, *J* = 11.2 Hz), H-13*β* with H-11 (*δ*_H_ 2.45, d, *J* = 10.8 Hz), and H-11 with H_3_-19 revealed the *β*-orientation of H-11. By contrast, H-2 did not exhibit NOE interaction with H-3, revealing the downward orientation of H-3. The NOE correlation of H-3 (*δ*_H_ 4.98, d, *J* = 10.4 Hz) with H-5 (*δ*_H_ 4.21, dd, *J* = 10.8, 5.2 Hz), H-5 with H-7 (*δ*_H_ 4.98, d, *J* = 10.8 Hz), H-7 with H-9*α* (*δ*_H_ 2.08, m), and H-9*α* with H_3_-20 (*δ*_H_ 1.28, s), suggesting the *α*-orientation of H-5 and H_3_-20. Further, the *E* geometry of C-3/C-4 and C-7/C-8 was determined from the upfield shifted methyl groups of C-18 (*δ*_C_ 10.3) and C-19 (*δ*_C_ 14.9). The *^3^J* values between H-2 and H-3 (10.4 Hz), H-5 and H-6*β* (10.8 Hz), and H-6*β* and H-7 (10.8 Hz) were found to be consistent with the relative configuration shown in Figure 3. In order to compare the NMR data with those of **2** and other isosarcophine-derived metabolites measured in C_6_D_6_ of our previous studies [13,14], the ^1^H and ^13^C NMR spectra of **1** were also measured, and the results (Table 1) can further confirm the structure of **1** was elucidated. The absolute configuration of **1** was determined by comparison the CD spectrum of **1** with that of isosarcophine (**3**), as shown in Figure 4. The CD spectrum of **1** showed the positive Cotton effect at 227.0 nm (Δε = +34.8) for the π–π* transition, while the negative Cotton effect at 245.5 nm (Δε = −15.6) for the n–π* transition. This evidence demonstrated the fact of 2*S*-configuration [27,28]. Due to the biogenesis of **1** from isosarcophine, the absolute configuration of **1** was defined as 2*S*,5*R*,11*R*,12*R*,3*E*,7*E*.

Cherbonolide N (**2**) was afforded as a colorless oil. Its HRESIMS data showed the sodiated ion peak at *m/z* 355.1880 [M + Na]^+^ (calculated 355.1879, C_20_H_28_O_4_Na), indicating the molecular formula of C_20_H_28_O_4_ and seven degrees of unsaturation. The IR spectrum showed the absorption peaks at 3481 and 1741 cm^−1^, suggesting the presence of the hydroxy group and ester group. In the ^13^C spectrum, **2** showed 20 carbon signals, assigning to three methyls, seven methylenes, four methines, and six quaternary carbons with the DEPT spectrum assistance. ^1^H and ^13^C NMR spectra revealed the signals of a carbonyl group (*δ*_C_ 172.3, C), a tetrasubstituted double bond (*δ*_C_ 166.1, C; 128.1, C), two trisubstituted double bonds (*δ*_H_ 4.50, d, *J* = 8.4 Hz; *δ*_C_ 120.7, CH; 139.2, C; *δ*_H_ 4.76, d, *J* = 10.4 Hz; *δ*_C_ 127.2, CH; 133.8, C), an oxygen-bearing quaternary carbon (*δ*_C_ 79.4, C), two oxygen-bearing methines (*δ*_H_ 4.93, d, *J* = 8.4 Hz; *δ*_C_ 79.2, CH; *δ*_H_ 3.21, dd, *J* = 10.4, 6.4 Hz; *δ*_C_ 70.7, CH), and an oxygen-bearing methylene (*δ*_H_ 4.46, d, *J* = 14.8 Hz; 4.31, dd, *J* = 14.8, 1.6 Hz; *δ*_C_ 55.9, CH_2_). 

The planar structure of **2** was also elucidated by the 2D NMR spectra. The COSY spectrum showed that compound **2** possesses four partial structures from H-2 to H-3, H_2_-5 via H_2_-6 to H-7, H_2_-9 via H_2_-10 to H-11, and H_2_-13 to H_2_-14. All of the partial structures were linked by HMBC correlations from H_2_-17 to C-1, C-15, C-16; H_3_-18 to C-3, C-4, C-5; H_3_-19 to C-7, C-8, C-9; H_3_-20 to C-11, C-12, C-13; and H_2_-13 and H_2_-14 to C-1. Further, the HMBC correlation from both H-2 (*δ*_H_ 4.93, d, *J* = 8.4 Hz) and H_2_-17 (*δ*_H_ 4.46, d, *J* = 14.8 Hz; 4.31, dd, *J* = 14.8, 1.6 Hz) to C-16 (*δ*_C_ 172.3) indicated the presence of an *α*-methylene-*α*, *β*-unsaturated-γ-lactone ring. Accordingly, **2** should possess an additional degree of unsaturation which could be an additional ring. The HMBC correlation from H_2_-17 to C-12 (*δ*_C_ 79.4) demonstrated the presence of an ether linkage between C-12 and C-17. Based on the above evidence, the structure of **2** was established.

**Figure 5:**
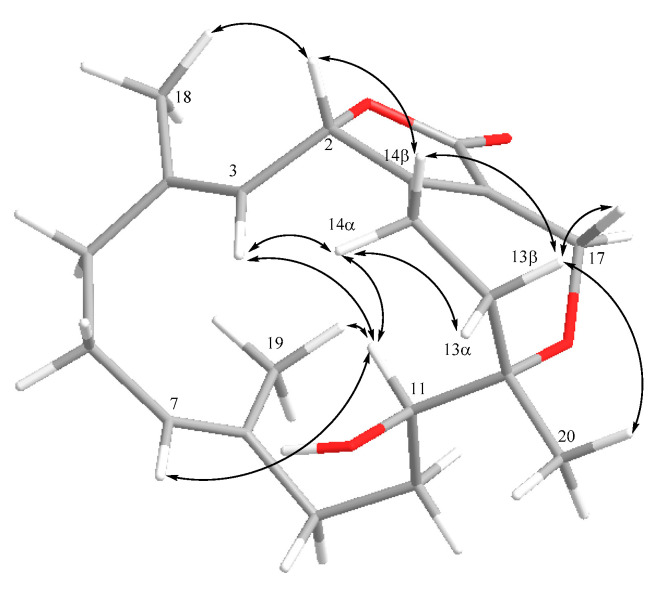
Selective NOE correlations of **2**.

Detailed analysis of the NOESY spectrum was applied for the elucidation of the relative configuration of **2** (Figure 5). It was found the NOE correlations between H-2 and H-14*β* (*δ*_H_ 1.66, m), H-14*β* and H-13*β* (*δ*_H_ 1.97, m), and also H-13*β* and H_3_-20 (*δ*_H_ 1.02, s), suggesting the *β*-orientation of H_3_-20. On the other hand, the NOE correlations between H-3 (*δ*_H_ 4.50, d, *J* = 8.4 Hz) and H-14*α* (*δ*_H_ 2.19, m), H-14*α* and H-11 (*δ*_H_ 3.21, dd, *J* = 10.4, 6.4 Hz), and H-11 and H-3 were observed, indicating the α-orientation of H-11 and the downward orientation of H-3. Furthermore, the upshift resonances of C-18 (*δ*_C_ 18.5) and C-19 (*δ*_C_ 15.2) demonstrated the *E* geometry of C-3/C-4 and C-7/C-8. The ^3^*J* values of H-2 and H-3 (8.4 Hz) and H-10 and H-11 (10.4 Hz) were also consistent with the relative configuration as shown in Figure 5. Based on the above evidence, the relative configuration of **2** was defined as 2S*,11R*,12S*,3*E*,7*E.*

**Figure 6:**
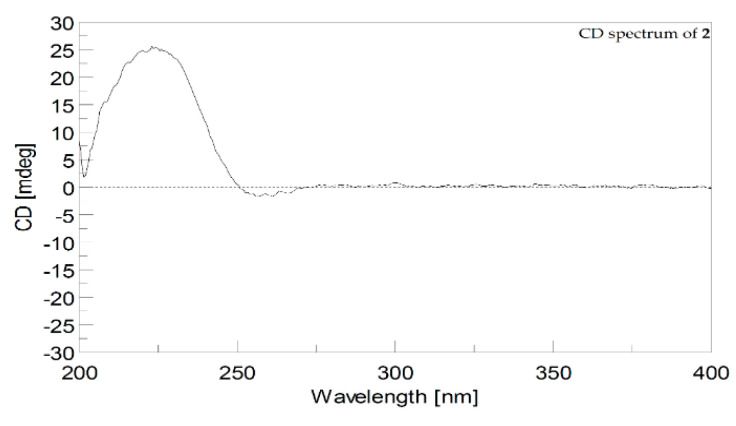
CD spectra (1.2 × 10^–4^ M, MeOH) of **2**.

**Scheme 1:**
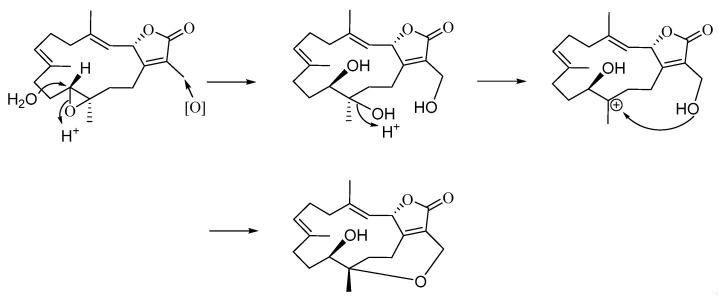
Proposed biosynthetic pathway of cherbonolide N (**2**).

In the present study, we isolated two cembranoids, cherbonolide M and N (**1** and **2**), from the soft coral *S. cherbonnieri.* Structurally, both isolates belong to the isosarcophine derivatives. By consideration of the chemical types of reported cembranoids, this is the first time to discover the cembranoidal scaffold possessing the unique tetrahydrooxepane with 12,17-ether linkage fusing with a γ-lactone. The plausible biosynthetic pathway was postulated, as shown in Scheme 1. Moreover, the CD spectrum of **2** (Figure 6) also revealed the positive effect at 225.0 nm (Δε = +25) and the negative effect at 255.4 nm (Δε = −2.0) as that of isosarcophine (**3**). Similar to compound **1**, metabolite **2** should also be biotransformed from isosarcophine and thus possessesed a (2*S*,11*R*,12*S*,3*E*,7*E*) absolute configuration.

In 1990, Kusumi et al. afforded isosarcophine from the Okinawan soft coral, *Sinularia mayi*, and demonstrated its moderate cytotoxicity against human colorectal carcinoma (HCT-116) cell line with the IC_50_ value of 64 μg/mL [25]. Subsequently, in 1992, Wu et al. also isolated the same metabolite from a Formosan soft coral, *Sarcophyton trocheliophorum*, which was also found to exhibit significant cytotoxicities toward human lung epithelial cells (A549), human colon carcinoma cells (HT-29), human oral epidermoid carcinoma cells (KB), mouse lymphoid neoplasm cells (P388), and promyelocytic leukemia cells (HL-60) with the ED_50_ values of 13.3, 16.9, 24.5, 0.7, and 6.7 μg/mL, respectively [26]. Many isosarcophine derivatives have been discovered from different marine soft corals and reported to display various biological activities [13,14,27]. 

For the discovery of bioactivities of new metabolites **1** and **2**, both compounds were examined according to the cytotoxic and anti-inflammatory activities. In the evaluation of cytotoxicity, both isolates were shown to be inactive against the proliferation of A549, DLD-1, and HuCCT-1 cell lines at the concentration of 30 μM. On the other hand, in anti-inflammatory assays at 30 μM, metabolite **2** showed inhibition of superoxide anion generation (20.6 ± 6.8%) and the elastase release (30.1 ± 3.3%)**,** while **1** only displayed 12.9 ± 5.7% and 16.7 ± 5.9% inhibition of superoxide anion generation and elastase release, respectively, in the fMLF/CB-induced human neutrophils. 

## 3. Experimental Section

### 3.1. General Experimental Procedures

Specific optical rotations of both compounds **1** and **2** were measured on a JASCO P-1020 polarimeter (JASCO Corporation, Tokyo, Japan), and their IR spectra were recorded on an FT/IR-4100 infrared spectrophotometer (JASCO Corporation, Tokyo, Japan). The low-resolution electrospray ionization mass spectrometry (LR-ESI-MS) and HR-ESI-MS experiments were carried out on a Bruker APEX II (Bruker, Bremen, Germany) mass spectrometer. The CD spectra of **1**–**3** were recorded by Jasco J-815 spectropolarimeter (JASCO, Tokyo, Japan) in MeOH. NMR spectra of **1** were acquired on a Varian Unity Inova 500 FT-NMR (Varian Inc., Palo Alto, CA, USA) at 500 MHz for ^1^H and 125 MHz for ^13^C in C_6_D_6_ at room temperature (25 °C). Further, NMR spectra of **1** and **2** were acquired on a Varian 400 MR FT-NMR (Varian Inc., Palo Alto, CA, USA) instrument at 400 MHz for ^1^H and 100 MHz for ^13^C in acetone-*d*_6_ and C_6_D_6_ at the same condition. Low-resolution electrospray ionization mass spectrometry (LRESIMS) and HRESIMS data were recorded on a Bruker APEX II (Bruker, Bremen, Germany) mass spectrometer. Normal-phase column chromatography was undertaken using silica gel (Merck, 230–400 mesh). Pre-coated silica gel plates (Merck, Kieselgel 60 F-254, 0.2 mm, Merck, Darmstadt, Germany) were used for analytical thin-layer chromatography (TLC). High-performance liquid chromatography was carried out for further purification of isolates, using a Hitachi L-2455 HPLC apparatus (Hitachi, Tokyo, Japan) with a Supelco C18 column (250 × 21.2 mm, 5 μm; Supelco, Bellefonte, PA, USA).

### 3.2. Animal Material

The collection and identification of the soft coral *S. cherbonnieri* were carried out as described previously [13,14].

### 3.3. Extraction and Isolation

The soft coral *S.* *cherbonnieri* (wet weight: 1.2 kg) was freeze-dried to remove the water. The dried sample was sliced into small pieces for EtOAc extraction. The combined EtOAc extract was concentrated under reduced pressure to afford an oily residue (10.2 g), which was chromatographed by a normal phase column with the gradient elution of acetone in *n*-hexane (0–100%) and methanol in acetone (0–100%) to separate 19 fractions. Fraction 13, eluting with *n*-hexane-acetone (1:1), was further purified over Sephadex LH-20 column using acetone to afford five subfractions (A–E). Subfraction 13-D was further purified by reversed-phase HPLC with the elution of acetonitrile-H_2_O (1.6:1, 5.0 mL/min) to afford **1** (7.1 mg, *t*_R_ 61.9 min). Subfraction 13-E was also purified by reversed-phase HPLC with the elution of acetonitrile-H_2_O (1.7:1, 5.0 mL/min) to afford **2** (3.6 mg, *t*_R_ 54.5 min).

Cherbonolide M (**1**): colorless amorphous oil, ${\left[\alpha \right]}_{D}^{25}$ +29.0 (*c* 1.00, CHCl_3_), IR (KBr) ν_max_ 3445, 2927, 2862, 1749, 1679, 1455, 1387, 1093, 1006, 755 cm^−1^; CD (1.2 × 10^–^^4^ M, MeOH) λ_max_ Δε 245.5 (−15.6), and 227.0 (+34.8) nm; for ^13^C and ^1^H data see Table 1; electrospray ionization mass spectrometry (ESIMS) *m/z* 355; HRESIMS *m/z* 355.1882 [M + Na]^+^ (calculated for C_20_H_28_O_4_Na: 355.1880).
Cherbonolide N (**2**): colorless amorphous oil, ${\left[\alpha \right]}_{D}^{25}$ +15.0 (*c* 1.00, CHCl_3_), IR (KBr) ν_max_ 3481, 2930, 1741, 1678, 1437, 1383, 1102, 1061, 986, 754 cm^−1^; CD (1.2 × 10^−4^ M, MeOH) λ_max_ Δε 255.4 (−2.0), and 225.0 (+25.0) nm; for ^13^C and ^1^H data see Table 1; ESIMS *m/z* 355; HRESIMS *m/z* 355.1880 [M + H]^+^ (calculated for C_20_H_28_O_4_Na: 355.1879).

### 3.4. Cytotoxicity Testing

To measure the cytotoxicities of **1** and **2**, three different concentrations of both compounds were added to A549, DLD-1, and HuCCT-1 cell lines for 72 h. The results were detected using the Alamar Blue assay [29,30]. 

### 3.5. Anti-Inflammatory Assay

The generation of the superoxide anion and elastase release in the fMLF/CB-induced primary human neutrophils was screened according to the previous description [13]. 

## 4. Conclusions

Two new isosarcophine derivatives, cherbonolides M (**1**) and N (**2**), were isolated from the continuous investigation of the soft coral *S.* *cherbonnieri*. Structurally, metabolite **2** is the first cembranoid possessing the unique cembranoidal scaffold of tetrahydrooxepane with the 12,17-ether linkage fusing with a *γ*-lactone. A plausible biosynthetic pathway was postulated for compound **2**. In the anti-inflammatory assay, compound **2** was found to show moderate activity in inhibiting the generation of superoxide anion and elastase release in the fMLF/CB-induced human neutrophils. Based on the above statements and our previous discoveries [13,14], the soft coral *S.* *cherbonnieri* was demonstrated to be an attractive source of bioactive diterpenoids. Further, it has been proven that the soft coral *S. glacum* existed in more than seven genetically distinct clades and led to the high structural diversification of natural products of this species [31]. Currently, over 300 cembranoids have been discovered from the *Sarcophyton* genera [1], which might also come from the same reason. According to our investigation and related studies of other research groups [13,14,32,33,34,35,36,37,38], the soft corals of genus *Sarcophyton* could be considered ideal organisms for discovering natural products with diversified structures and biological activities.

## Figures and Tables

**Table 1:** ^13^C and ^1^H NMR spectroscopic data of **1** and **2**.

Position	1 ^α^		1 ^b^		2 ^c^	
	*δ*_C_	*δ*_H_	*δ*_C_	*δ*_H_	*δ*_C_	*δ*_H_
1	162.4 (C)		159.9 (C)		166.1 (C)	
2	78.4 (CH) ^d^	5.64 d (10.4) ^e^	77.4 (CH)	4.99 d (10.5)	79.2 (CH)	4.93 d (8.4)
3	123.4 (CH)	4.98 d (10.4)	123.8 (CH)	4.55 d (10.0)	120.7 (CH)	4.50 d (8.4)
4	147.5 (C)		145.5 (C)		139.2 (C)	
5	77.5 (CH)	4.21 dd (10.8, 5.2)	77.3 (CH)	3.77 dd (10.5, 5.5)	36.9 (CH_2_)	1.66 m; 1.87 m
6	33.8 (CH_2_)	2.28 m; 2.52 m	32.8 (CH_2_)	2.23 m; 2.31 m	24.3 (CH_2_)	1.77 m; 2.27 m
7	122.5 (CH)	4.98 d (10.8)	121.4 (CH)	4.55 d (10.0)	127.2 (CH)	4.76 d (10.4)
8	134.9 (C)		134.4 (C)		133.8 (C)	
9	37.3 (CH_2_)	2.08 m; 2.31 m	36.9 (CH_2_)	1.70 m; 1.98 m	36.1 (CH_2_)	1.92 m; 1.94 m
10	24.5 (CH_2_)	2.07 m; 1.23 m	24.2 (CH_2_)	1.06 m; 1.90 m	25.8 (CH_2_)	0.95 m; 1.98 m
11	62.0 (CH)	2.45, d (10.8)	61.1 (CH)	2.28 dd (10.5, 2.5)	70.7 (CH)	3.21 dd (10.4, 6.4)
12	61.1 (C)		59.9 (C)		79.4 (C)	
13	37.8 (CH_2_)	2.05 m; 1.04 t (11.2)	37.3 (CH_2_)	0.77 td (13.5, 2.5); 1.61 dd (13.0, 5.5)	29.4 (CH_2_)	1.70 m; 1.97 m
14	24.7 (CH_2_)	2.02 m; 2.71 m	23.8 (CH_2_)	1.53 d (13.5); 1.95 m	24.0 (CH_2_)	1.66 m; 2.19 m
15	123.5 (C)		123.8 (C)		128.1 (C)	
16	174.6 (C)		173.7 (C)		172.3 (C)	
17	8.6 (CH_3_)	1.78 s	8.7 (CH_3_)	1.67 s	55.9 (CH_2_)	4.31 dd (14.8, 1.6); 4.46 d (14.8)
18	10.3 (CH_3_)	1.74 s	9.8 (CH_3_)	1.33 s	18.5 (CH_3_)	1.27 s
19	14.9 (CH_3_)	1.72 s	14.5 (CH_3_)	1.31 s	15.2 (CH_3_)	1.36 s
20	15.9 (CH_3_)	1.28 s	15.9 (CH_3_)	1.02 s	22.5 (CH_3_)	1.02 s

^α^ ^13^C and ^1^H spectroscopic data of **1** recorded at 100 and 400 MHz in acetone-*d_6_*. ^b 13^C and ^1^H spectroscopic data of **1** recorded at 125 and 500 MHz in C_6_D_6_. ^c 13^C and ^1^H spectroscopic data of **2** recorded at 100 and 400 MHz in C_6_D_6_. ^d^ Attached protons were deduced from DEPT experiments. ^e^ *J* values (in Hz) in parentheses.

## Data Availability

Data available in a publicly accessible repository.

## Supplementary Materials

The following are available online at https://www.mdpi.com/article/10.3390/md19050260/s1, Figure S1: ESIMS spectrum of **1**; Figure S2: HRESIMS spectrum of **1**; Figure S3: IR spectrum of **1**; Figure S4: CD spectrum (1.2 × 10^−4^ M, MeOH) of **1**; Figure S5: ^1^H NMR spectrum of **1** in acetone-*d*_6_ at 400 MHz; Figure S6: ^1^H NMR spectrum (from 0.9 to 2.9 ppm) of **1** in acetone-*d*_6_ at 400 MHz; Figure S7: ^13^C NMR spectrum of **1** in acetone-*d*_6_ at 100 MHz; Figure S8: DEPT spectrum of **1** in acetone-*d*_6_; Figure S9: HSQC spectrum of **1** in acetone-*d*_6_; Figure S10: COSY spectrum of **1** in acetone-*d*_6_; Figure S11: HMBC spectrum of **1** in acetone-*d*_6_; Figure S12: NOESY spectrum of **1** in acetone-*d*_6_; Figure S13: ^1^H NMR spectrum of **1** in C_6_D_6_ at 500 MHz; Figure S14: ^1^H NMR spectrum (from 0.6 to 2.5 ppm) of **1** in C_6_D_6_ at 500 MHz; Figure S15: ^13^C NMR spectrum of **1** in C_6_D_6_ at 125 MHz; Figure S16: DEPT spectrum of **1** in C_6_D_6_; Figure S17: HSQC spectrum of **1** in C_6_D_6_; Figure S18: COSY spectrum of **1** in C_6_D_6_; Figure S19: HMBC spectrum of **1** in C_6_D_6_; Figure S20: NOESY spectrum of **1** in C_6_D_6_; Figure S21: ESIMS spectrum of **2**; Figure S22: HRESIMS spectrum of **2**; Figure S23: IR spectrum of **2**; Figure S24: CD spectrum (1.2 × 10^−4^ M, MeOH) of **2**; Figure S25: ^1^H NMR spectrum of **2** in C_6_D_6_ at 400 MHz; Figure S26: ^1^H NMR spectrum (from 0.8 to 3.2 ppm) of **2** in C_6_D_6_ at 400 MHz; Figure S27: ^13^C NMR spectrum of **2** in C_6_D_6_ at 100 MHz; Figure S28: DEPT spectrum of **2**; Figure S29: HSQC spectrum of **2**; Figure S30: COSY spectrum of **2**; Figure S31: HMBC spectrum of **2**; Figure S32: NOESY spectrum of **2**. Figure S33: CD spectrum (1.6 × 10^−4^ M, MeOH) of isosarcophine (**3**).
