# Supplementary material for: Cherbonolides M and N from a Formosan Soft Coral Sarcophyton cherbonnieri
Source: Mar Drugs. 2021 May 1;19(5):260. doi: 10.3390/md19050260 (PMC8170881; doi:10.3390/md19050260)
Supplement: Supplementary file 1 [file marinedrugs-19-00260-s001.zip › marinedrugs-1157111-supplementary.pdf]

Cherbonolides M and N from a Formosan soft coral *Sarcophyton cherbonnieri*

Chia-Chi Peng [1], Tzu-Yin Huang [2], Chiung-Yao Huang [1], Tsong-Long Hwang [3,4,5], and Jyh-Horng Sheu [1,2,6,7,*]

[1] Department of Marine Biotechnology and Resources, National Sun Yat-sen University, Kaohsiung 804, Taiwan; Chia-Chi.Peng@hki-jena.de (C.-C.P.); huangcy@mail.nsysu.edu.tw (C.-Y.H.)
[2] Doctoral Degree Program in Marine Biotechnology, National Sun Yat-sen University, Kaohsiung 804, Taiwan; HuangTY@g-mail.nsysu.edu.tw (T.-Y. H.)
[3] Graduate Institute of Natural Products, College of Medicine, Chang Gung University, Taoyuan 333, Taiwan; htl@mail.cgu.edu.tw (T.-L. H.)
[4] Research Center for Industry of Human Ecology and Graduate Institute of Health Industry Technology, Chang Gung University of Science and Technology, Taoyuan 333, Taiwan
[5] Department of Anesthesiology, Chang Gung Memorial Hospital, Taoyuan 333, Taiwan
[6] Graduate Institute of Natural Products, Kaohsiung Medical University, Kaohsiung 807, Taiwan
[7] Department of Medical Research, China Medical University Hospital, China Medical University, Taichung 404, Taiwan
* Correspondence: sheu@mail.nsysu.edu.tw; Tel.: +886-7-525-2000 (ext. 5030); Fax: +886-7-525-5020

List of Figures

Figure S1: ESIMS spectrum of **1** 1

Figure S2: HRESIMS spectrum of **1** 1

Figure S3: IR spectrum of **1** 2

Figure S4: CD spectrum (1.2×10^{-4} M, MeOH) of **1** 2

Figure S5: ^{1}H NMR spectrum of **1** in acetone-d_6 at 400 MHz 3

Figure S6: ^{1}H NMR spectrum (from 0.9 to 2.9 ppm) of **1** in acetone-d_6 at 400 MHz 4

Figure S7: ^{13}C NMR spectrum of **1** in acetone-d_6 at 100 MHz 5

Figure S8: DEPT spectrum of **1** in acetone-d_6 6

Figure S9: HSQC spectrum of **1** in acetone-d_6 7

Figure S10: COSY spectrum of **1** in acetone-d_6 8

Figure S11: HMBC spectrum of **1** in acetone-d_6 9

Figure S12: NOESY spectrum of **1** in acetone-d_6 10

Figure S13: ^{1}H NMR spectrum of **1** in C_6D_6 at 500 MHz 11

Figure S14: ^{1}H NMR spectrum (from 0.6 to 2.5 ppm) of **1** in C_6D_6 at 500 MHz 12

Figure S15: ^{13}C NMR spectrum of **1** in C_6D_6 at 125 MHz 13

Figure S16: DEPT spectrum of **1** in C_6D_6 14

Figure S17: HSQC spectrum of **1** in C_6D_6 15

Figure S18: COSY spectrum of **1** in C_6D_6 16

Figure S19: HMBC spectrum of **1** in C_6D_6 17

Figure S20: NOESY spectrum of **1** in C_6D_6 18

Figure S21: ESIMS spectrum of **2** 19

Figure S22: HRESIMS spectrum of **2** 19

Figure S23: IR spectrum of **2** 20

Figure S24: CD spectrum (1.2×10^{-4} M, MeOH) of **2** 20

Figure S25: ^{1}H NMR spectrum of **2** in C_6D_6 at 400 MHz 21

Figure S26: ^{1}H NMR spectrum (from 0.8 to 3.2 ppm) of **2** in C_6D_6 at 400 MHz 22

Figure S27: ^{13}C NMR spectrum of **2** in acetone-d_6 at 100 MHz 23

Figure S28: DEPT spectrum of **2** 24

Figure S29: HSQC spectrum of **2** 25

Figure S30: COSY spectrum of **2** 26

Figure S31: HMBC spectrum of **2** 27

Figure S32: NOESY spectrum of **2** 28

Figure S33: CD spectrum (1.6 × 10^{-4} M, MeOH) of isosarcophine (**3**) 29

Spectroscopic data of cherbonolide M (**1**)

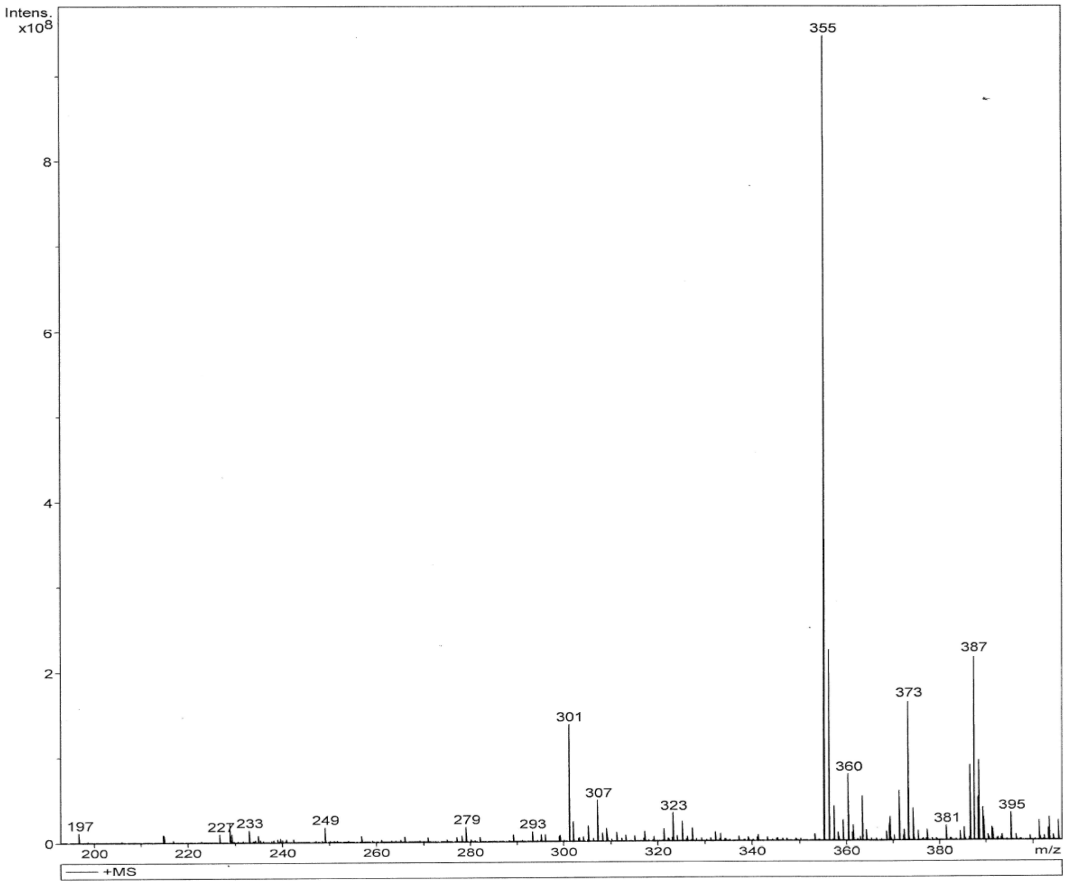

Figure S1: ESIMS spectrum of **1**

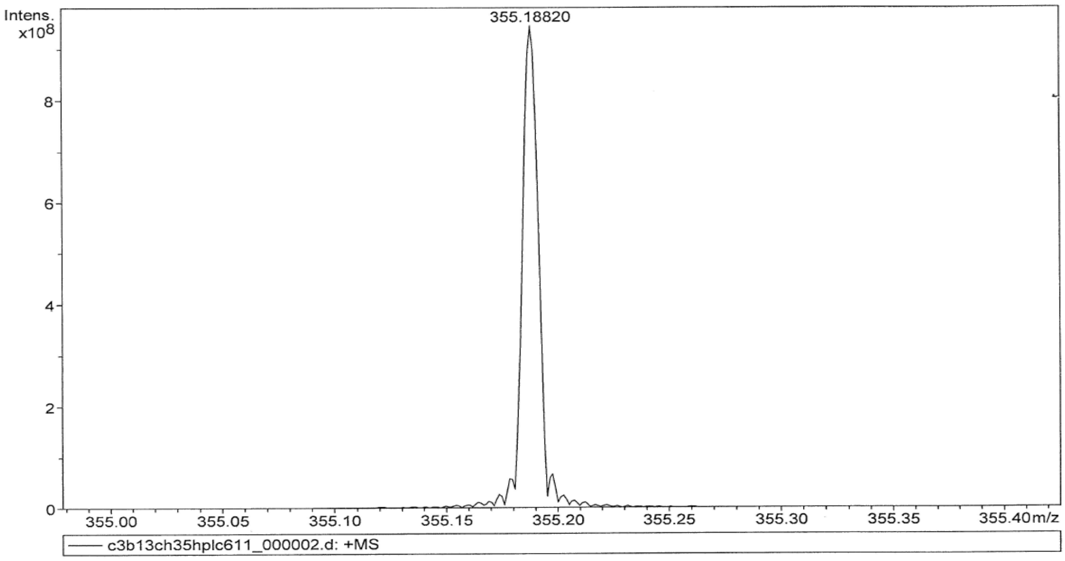

Meas. m/z	#	Formula	Score	m/z	err [mDa]	err [ppm]	mSigma	rdb	e⁻ Conf	N-Rule
355.18820	1	C 20 H 28 Na O 4	100.00	355.18798	-0.22	-0.63	14.9	6.5	even	ok

Figure S2: HRESIMS spectrum of **1**

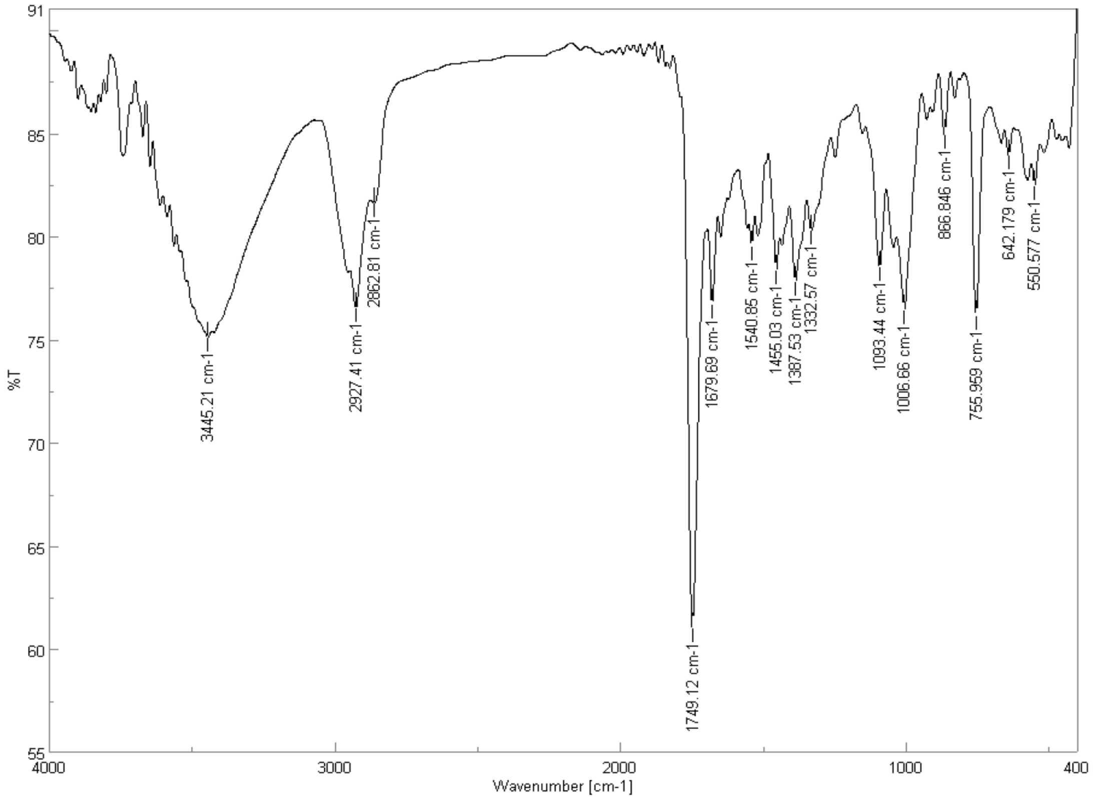

Figure S3: IR spectrum of **1**

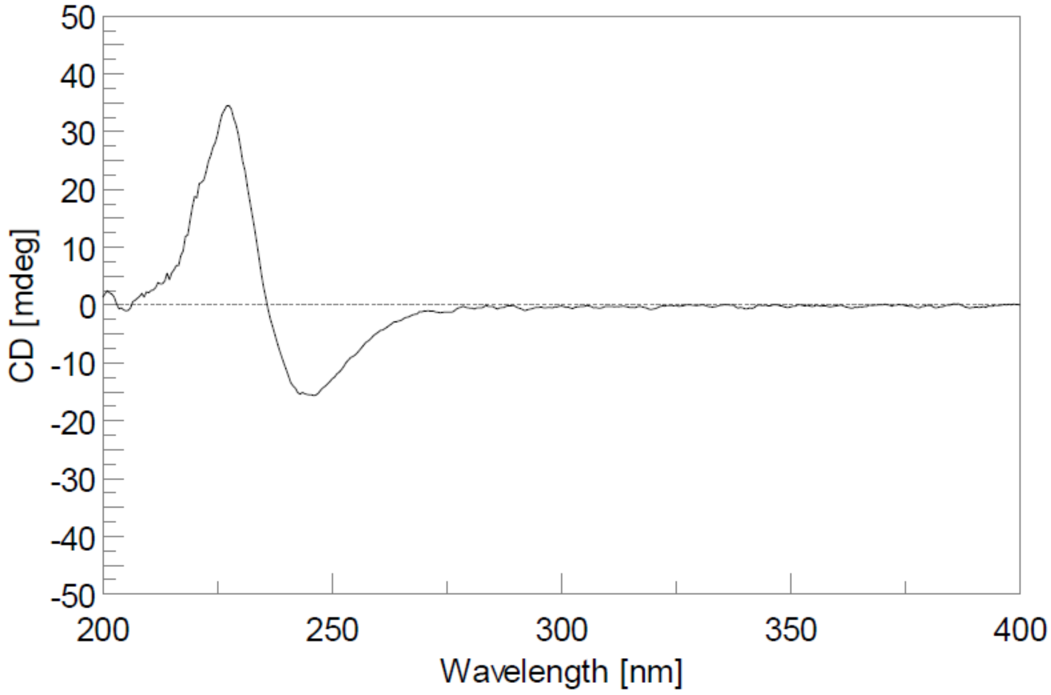

Figure S4: CD spectrum (1.2×10^{-4} M, MeOH) of **1**

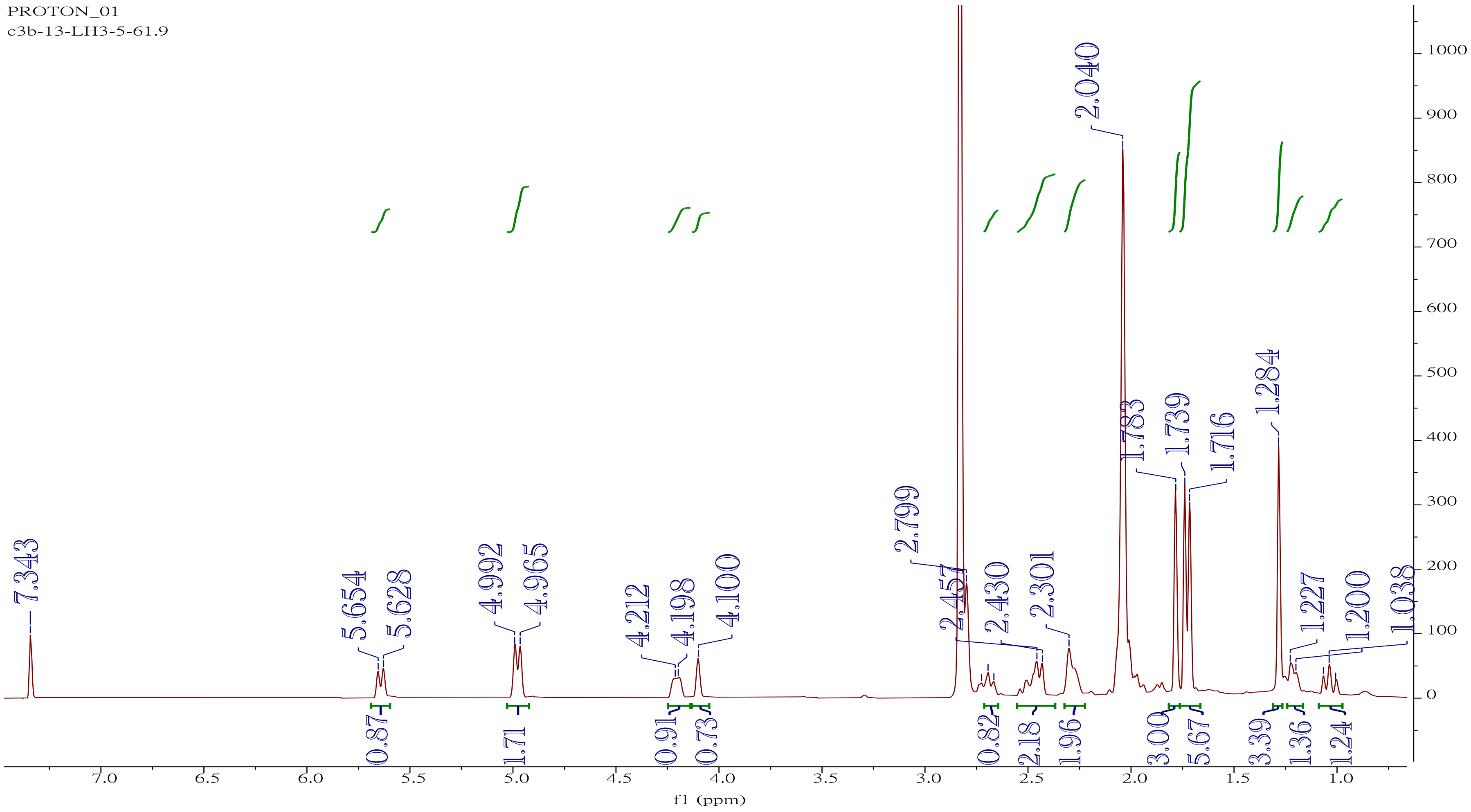

Figure S5: ^{1}H NMR spectrum of **1** in acetone-d_6 at 400 MHz

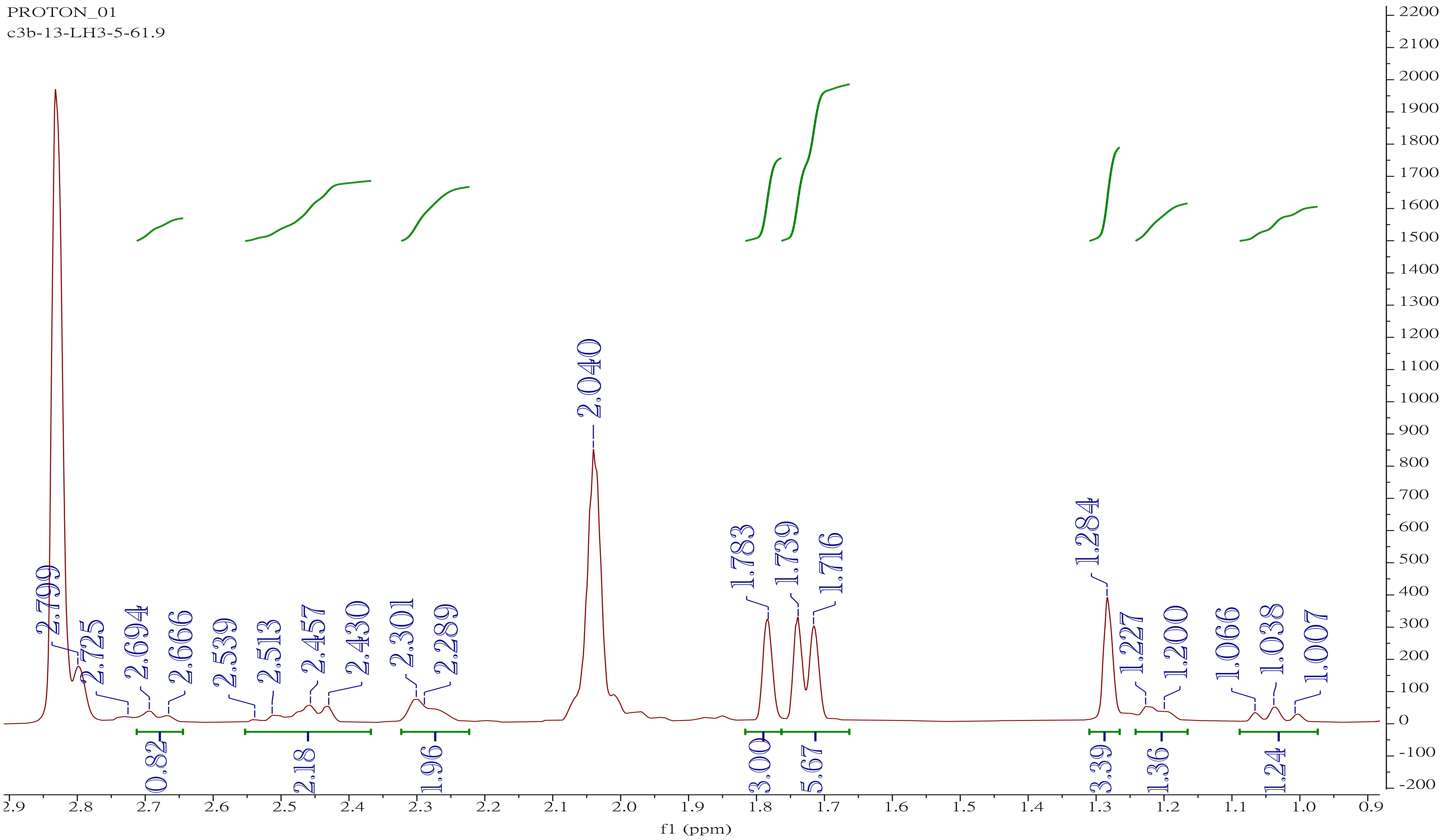

Figure S6: ^{1}H NMR spectrum (from 0.9 to 2.9 ppm) of **1** in acetone-d_6 at 400 MHz

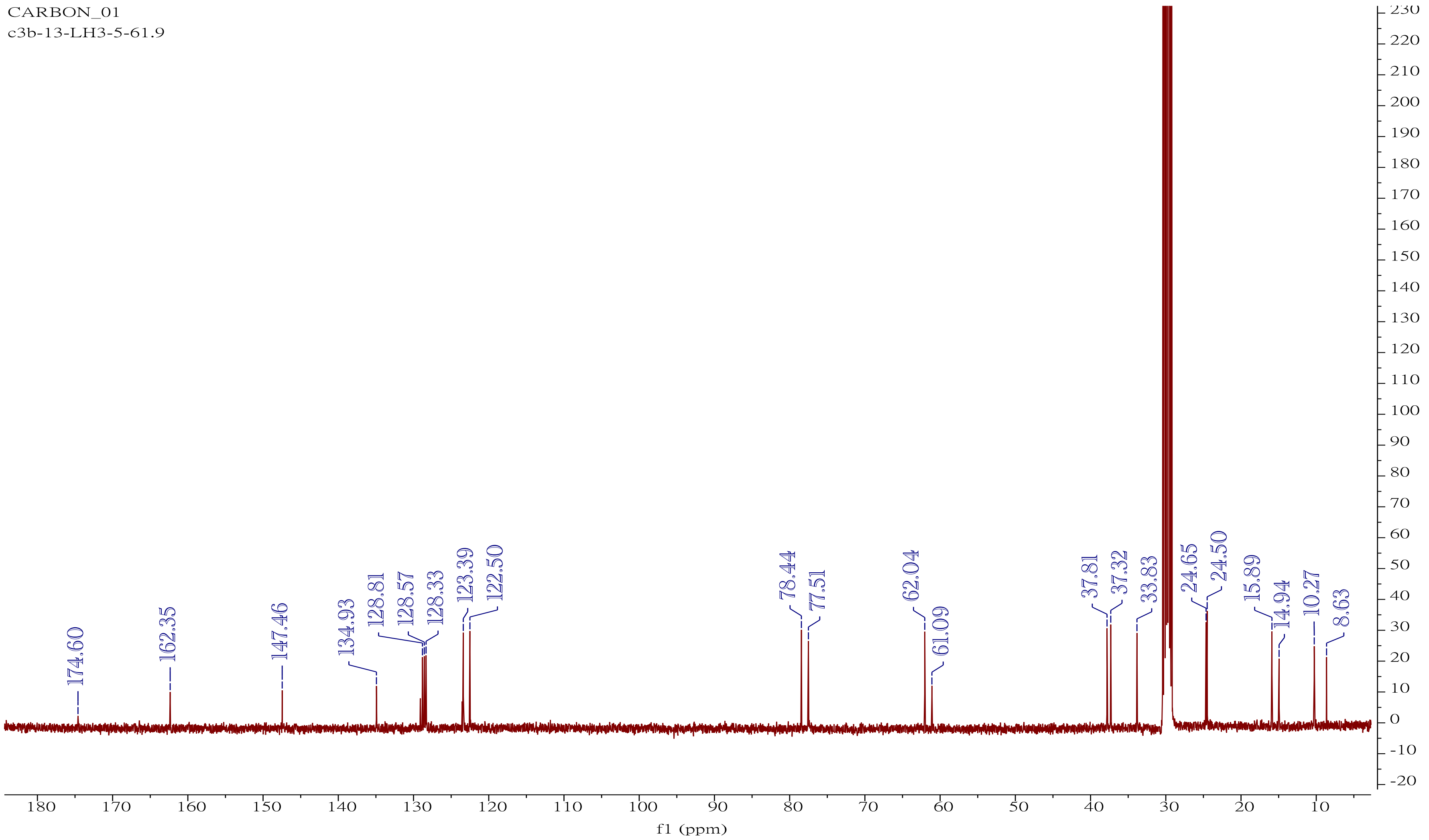

Figure S7: ^{13}C NMR spectrum of **1** in acetone-d_6 at 100 MHz

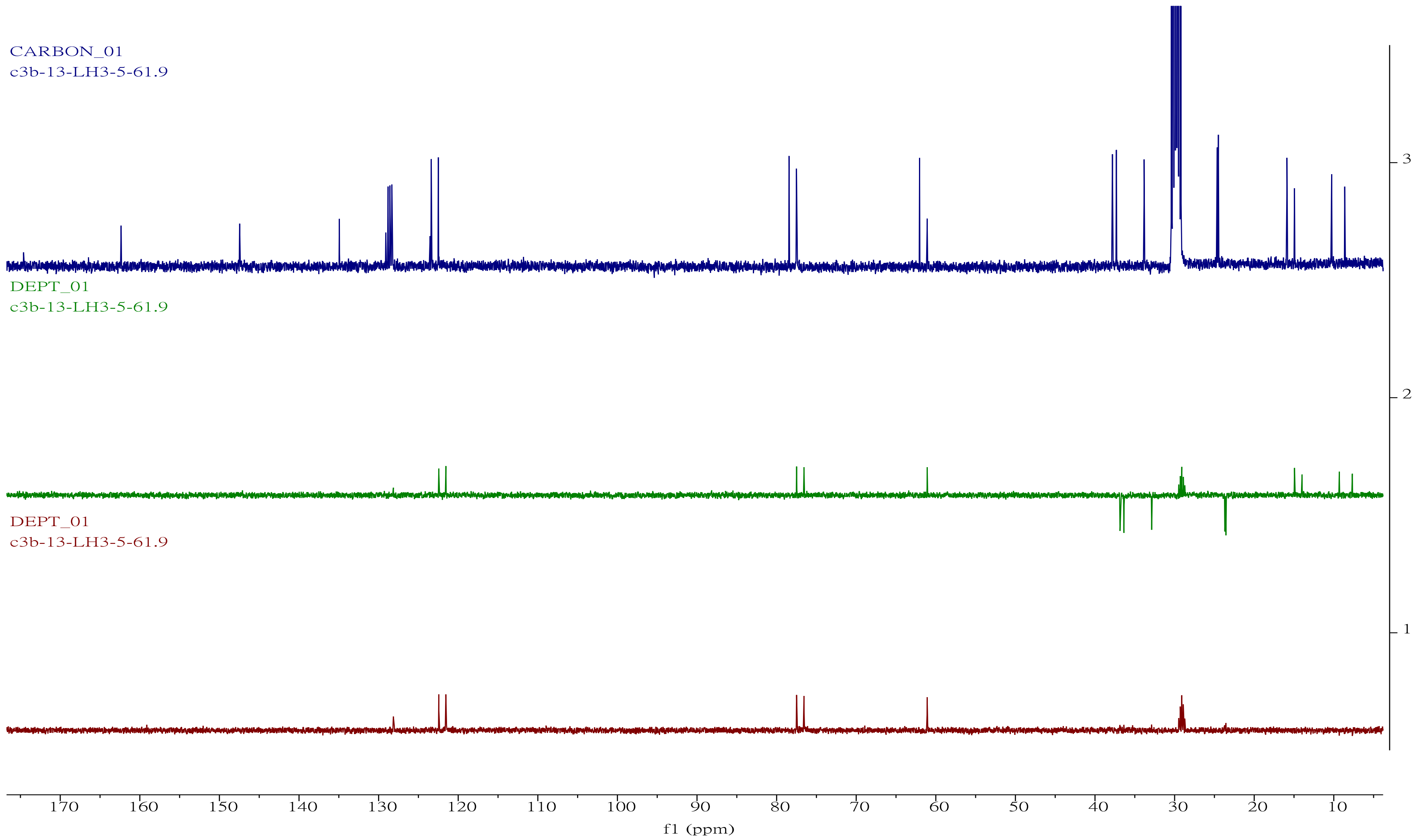

Figure S8: DEPT spectrum of **1** in acetone-d_6

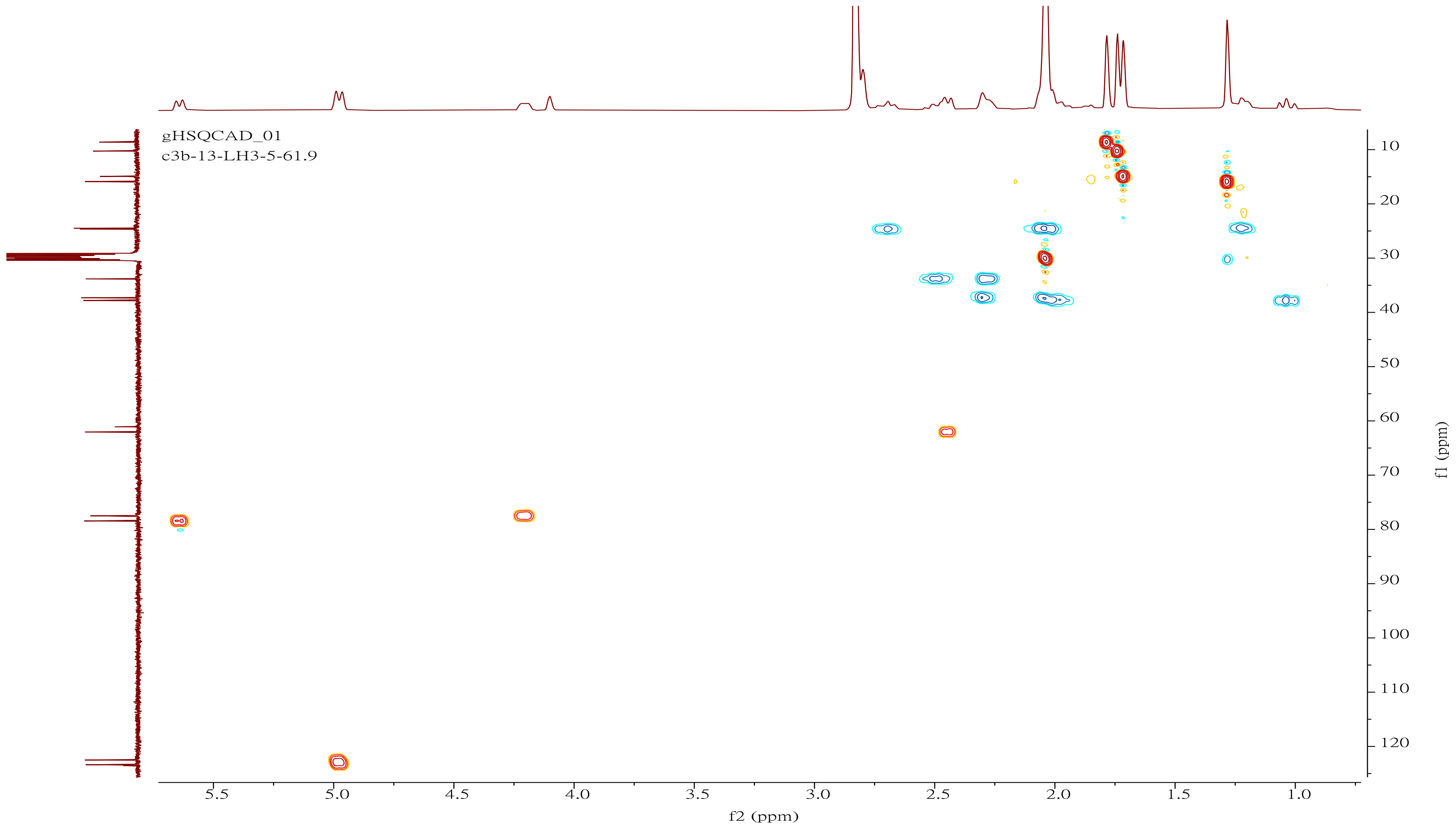

Figure S9: HSQC spectrum of **1** in acetone-d_6

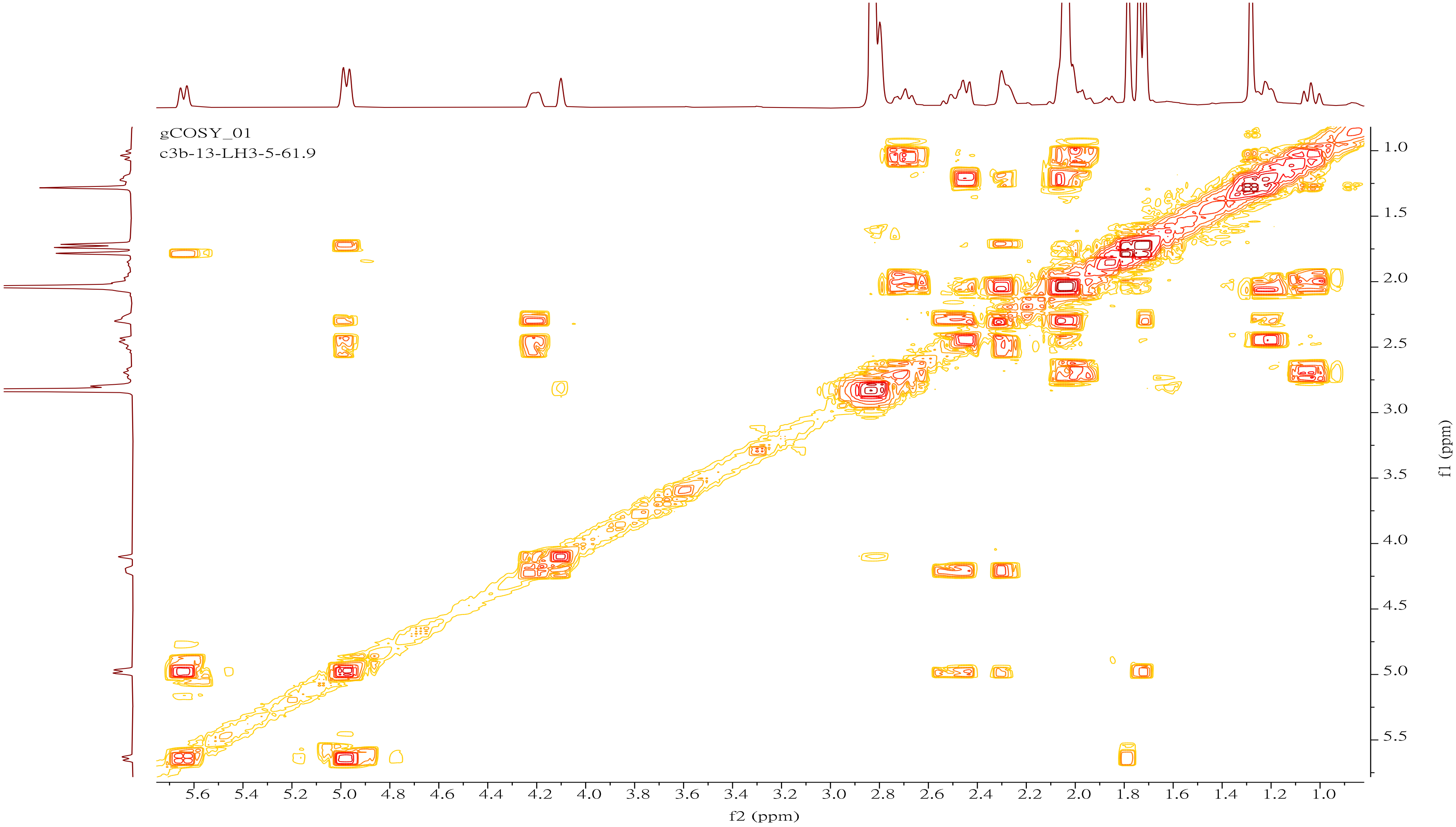

Figure S10: COSY spectrum of **1** in acetone-d_6

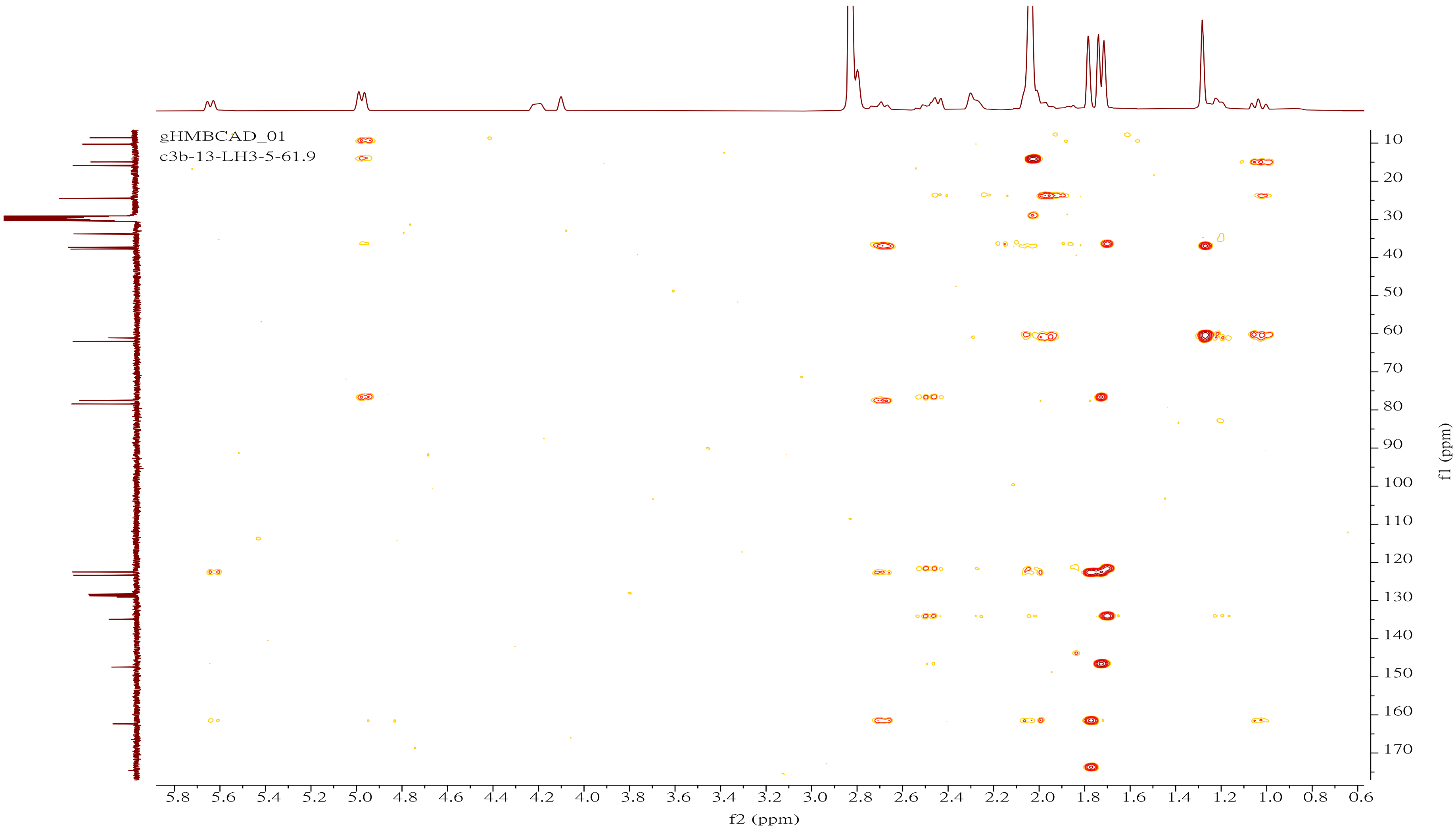

Figure S11: HMBC spectrum of **1** in acetone-d_6

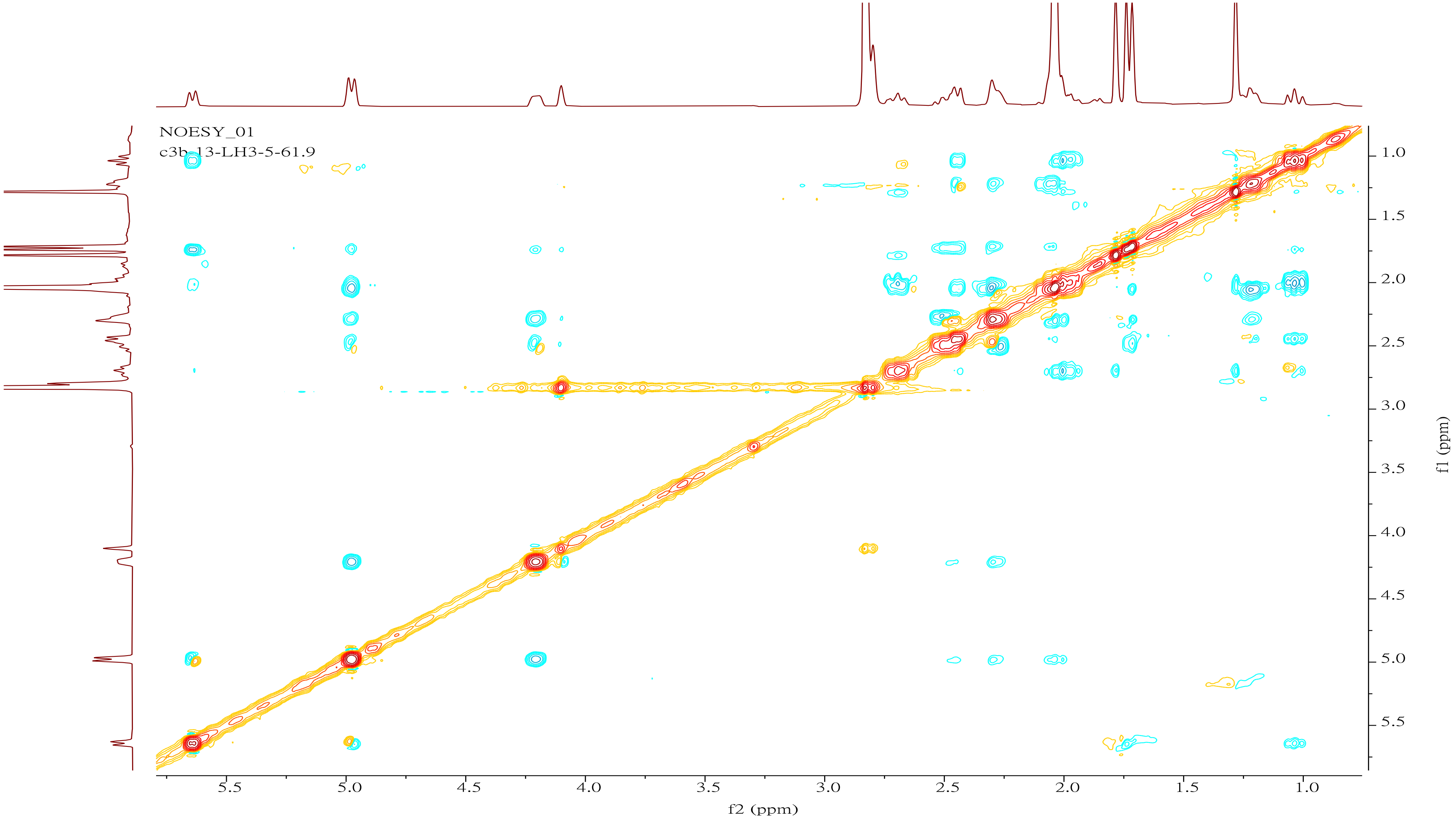

Figure S12: NOESY spectrum of **1** in acetone-d_6

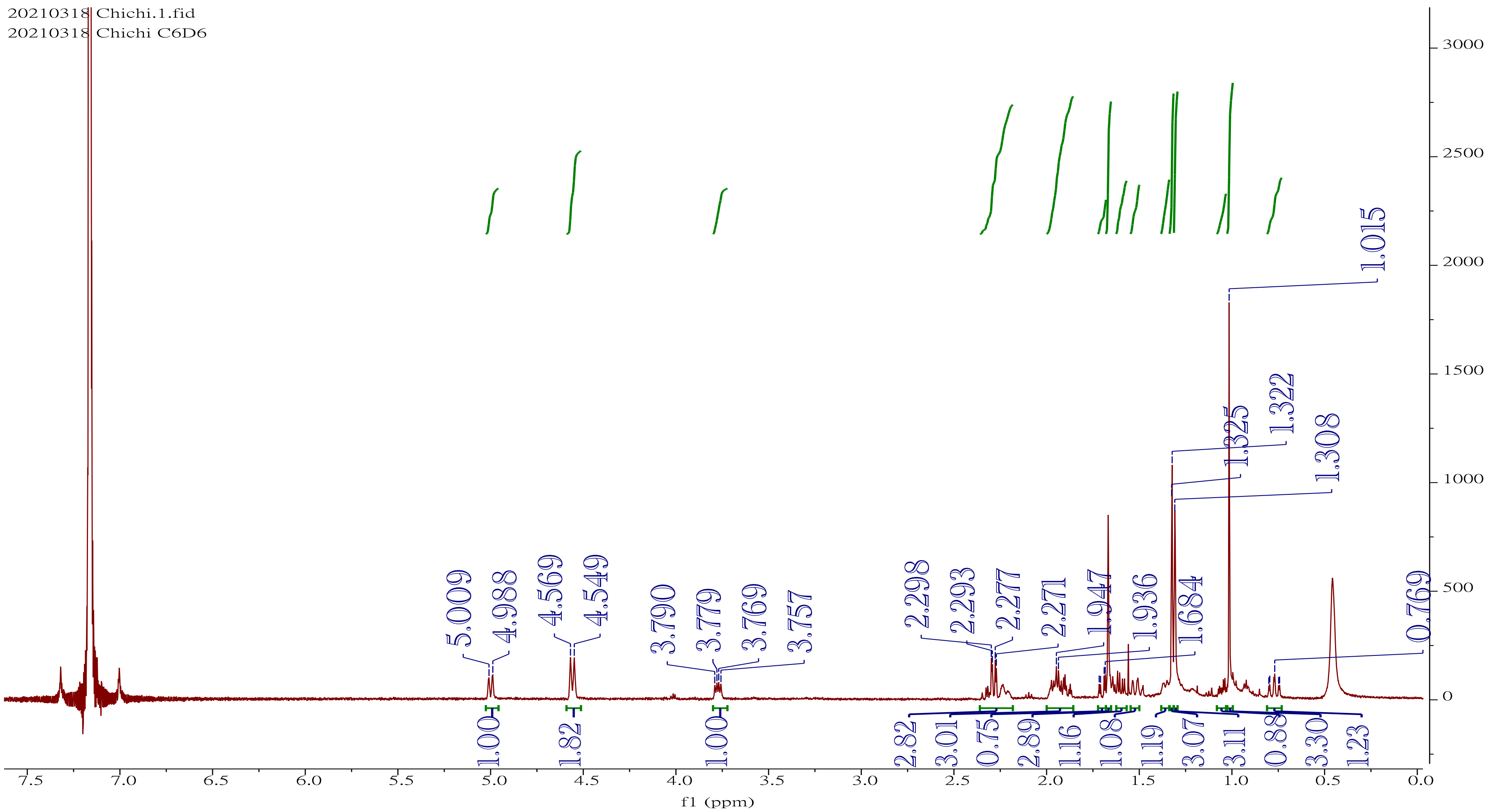

Figure S13: ^{1}H NMR spectrum of **1** in C_6D_6 at 500 MHz

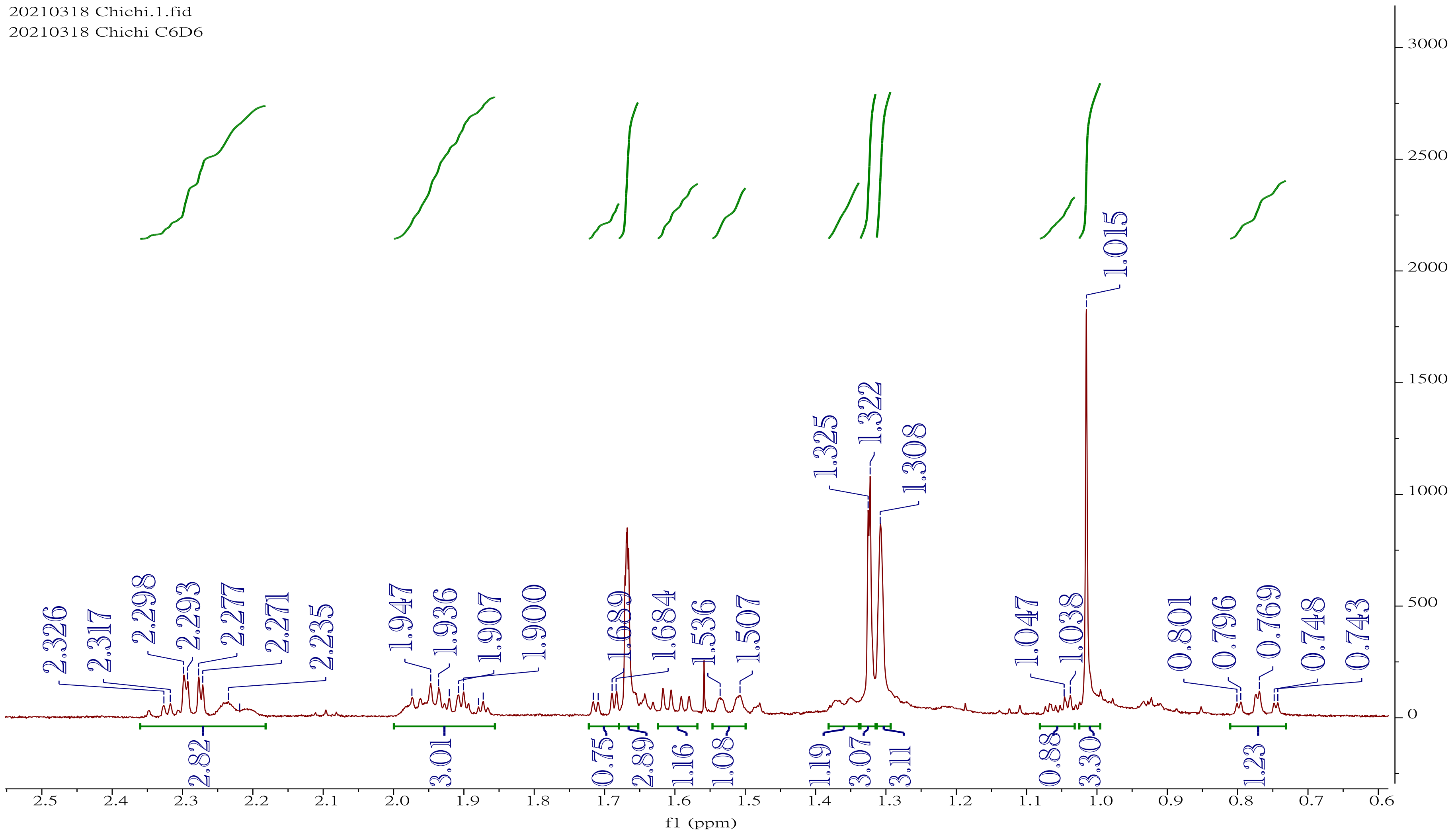

Figure S14: ^{1}H NMR spectrum (from 0.6 to 2.5 ppm) of **1** in C_6D_6 at 500 MHz

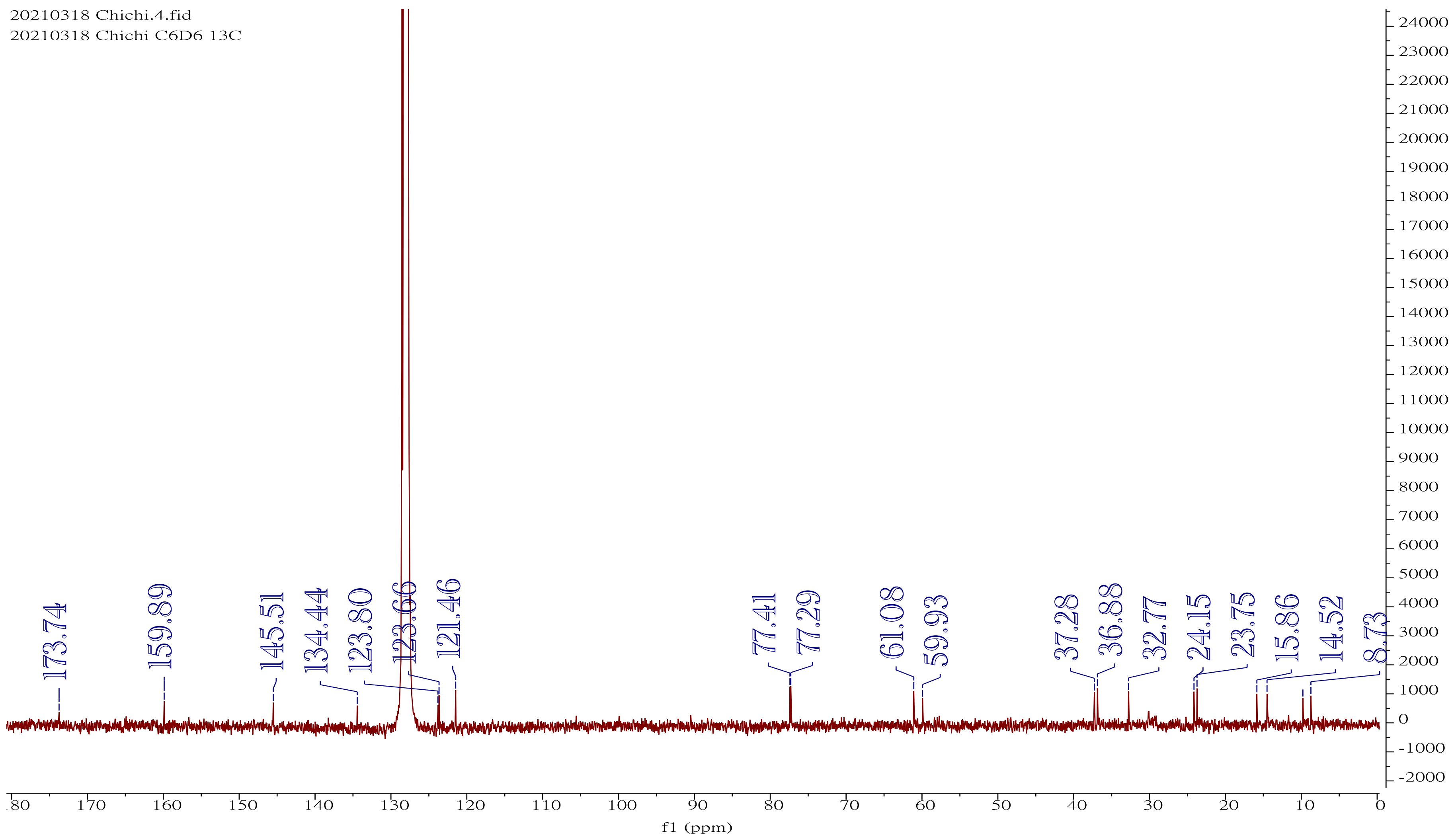

Figure S15: ^{13}C NMR spectrum of **1** in C_6D_6 at 125 MHz

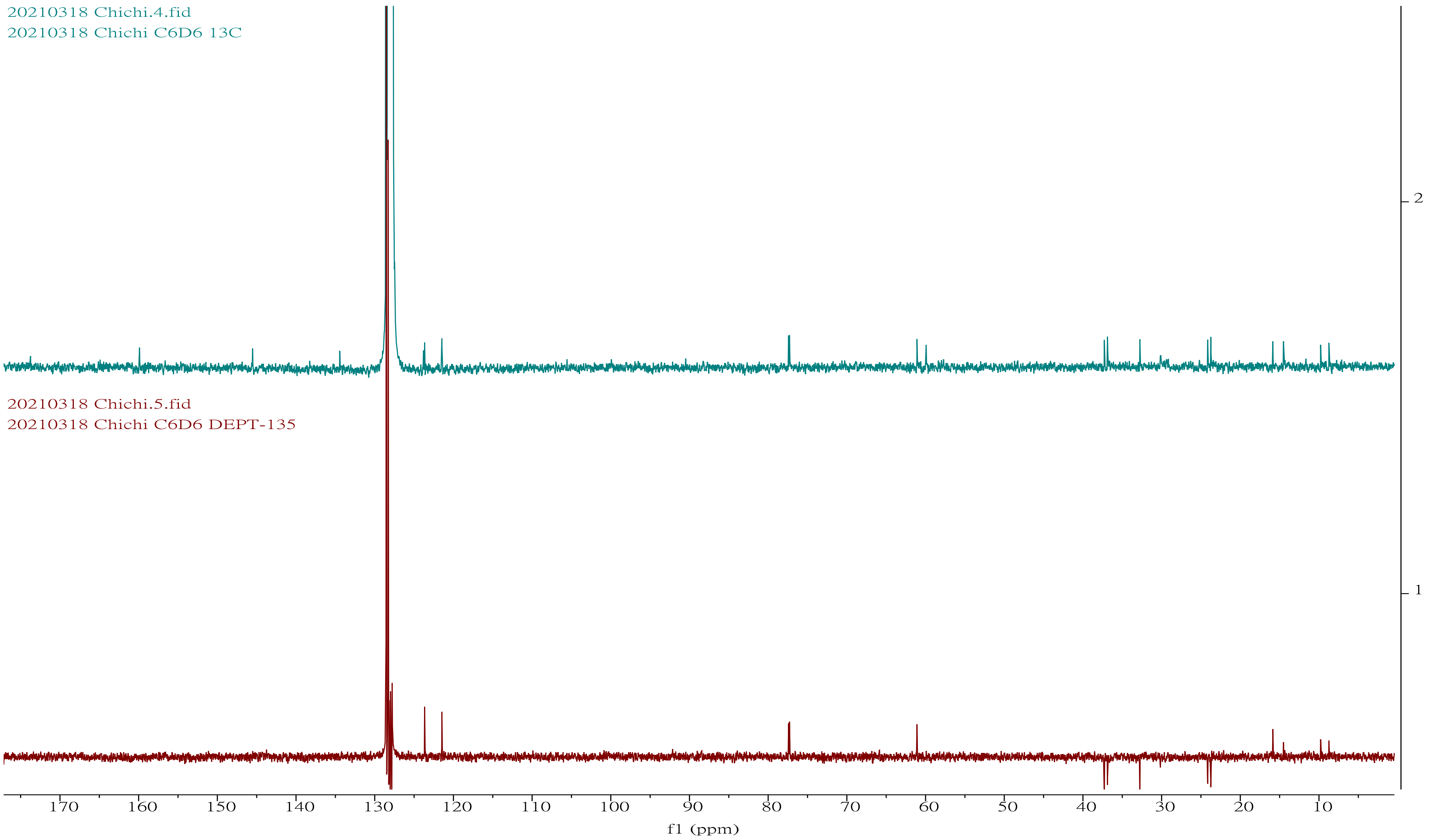

Figure S16: DEPT spectrum of **1** in C_6D_6

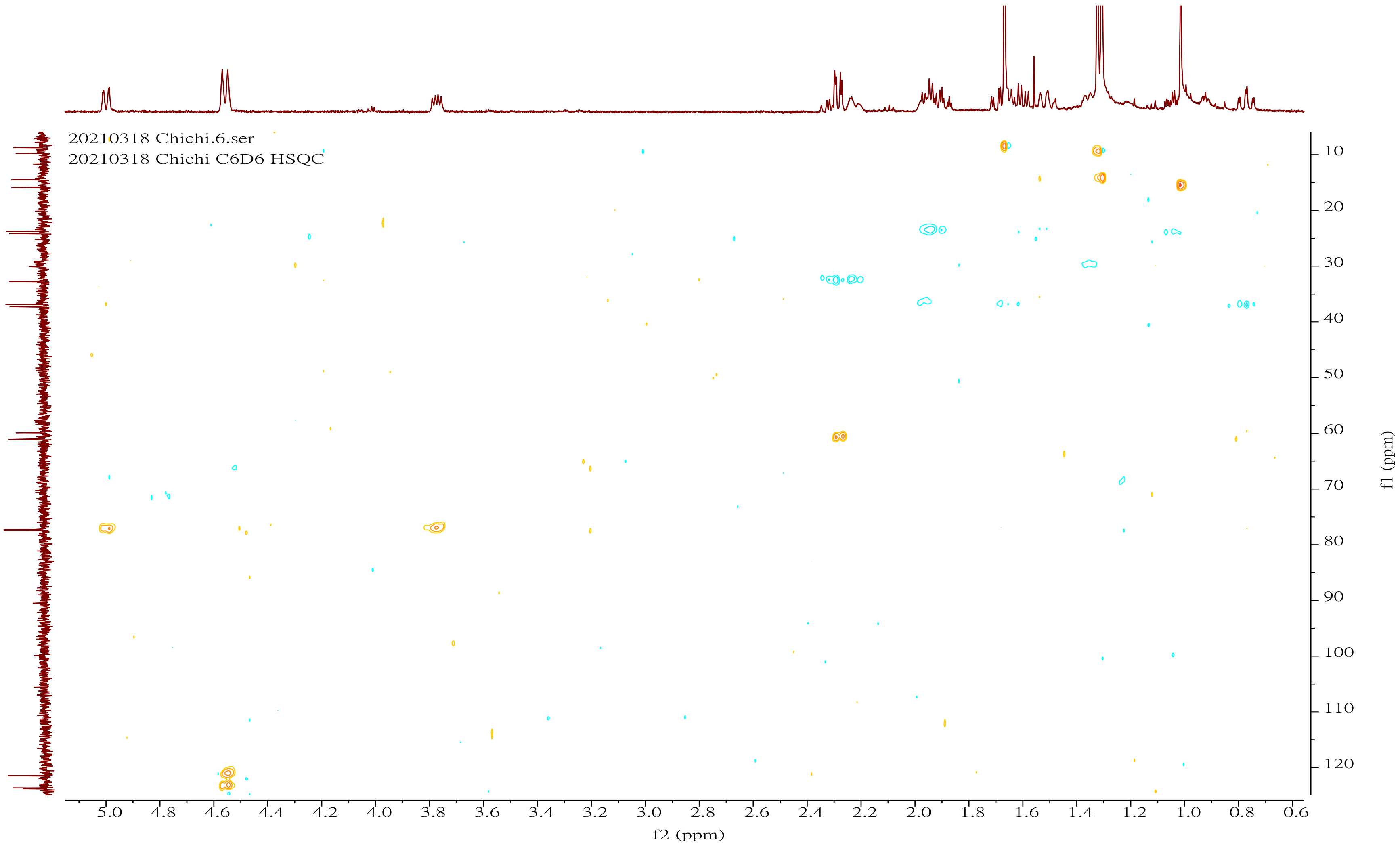

Figure S17: HSQC spectrum of **1** in C_6D_6

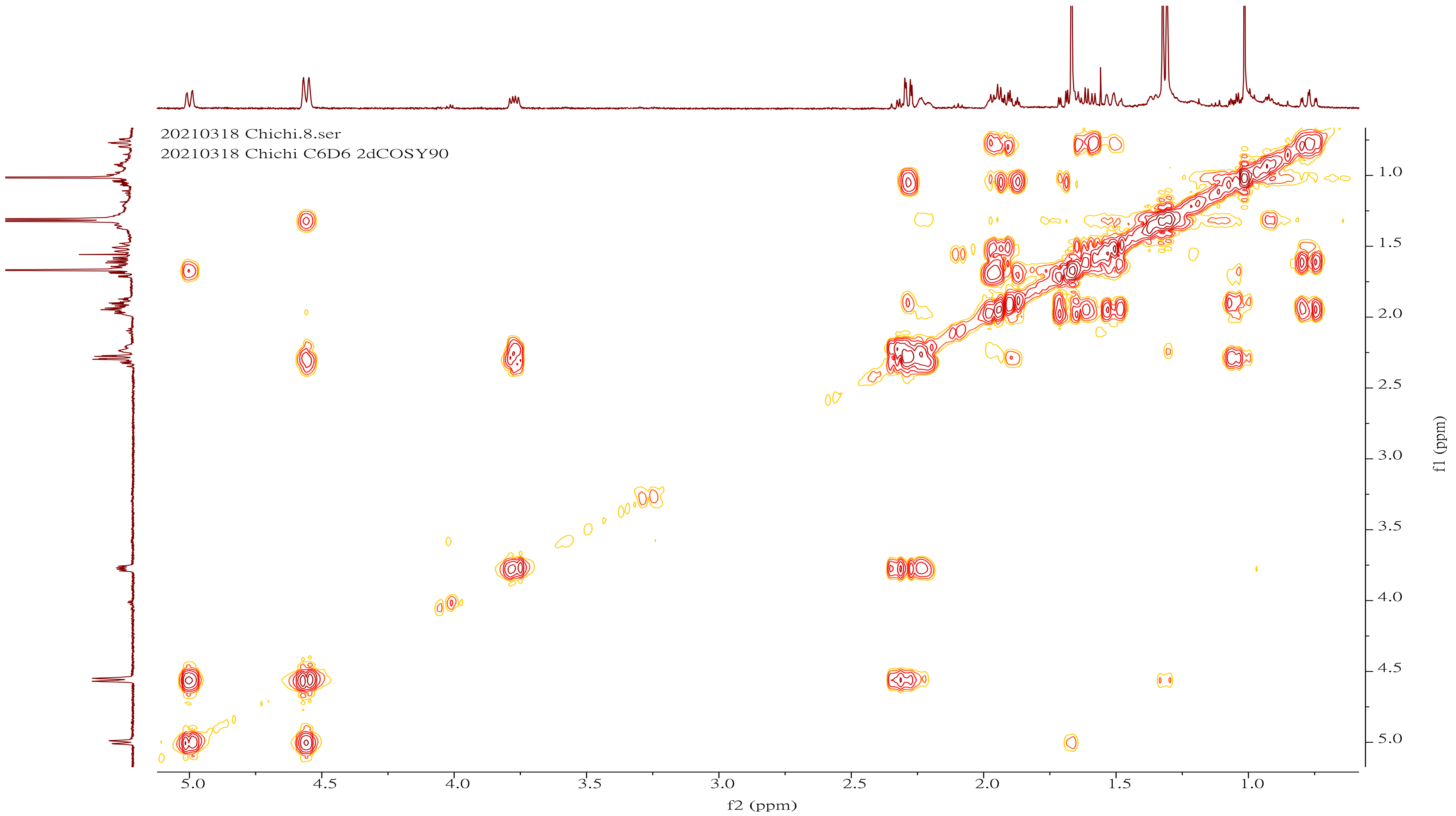

Figure S18: COSY spectrum of **1** in C_6D_6

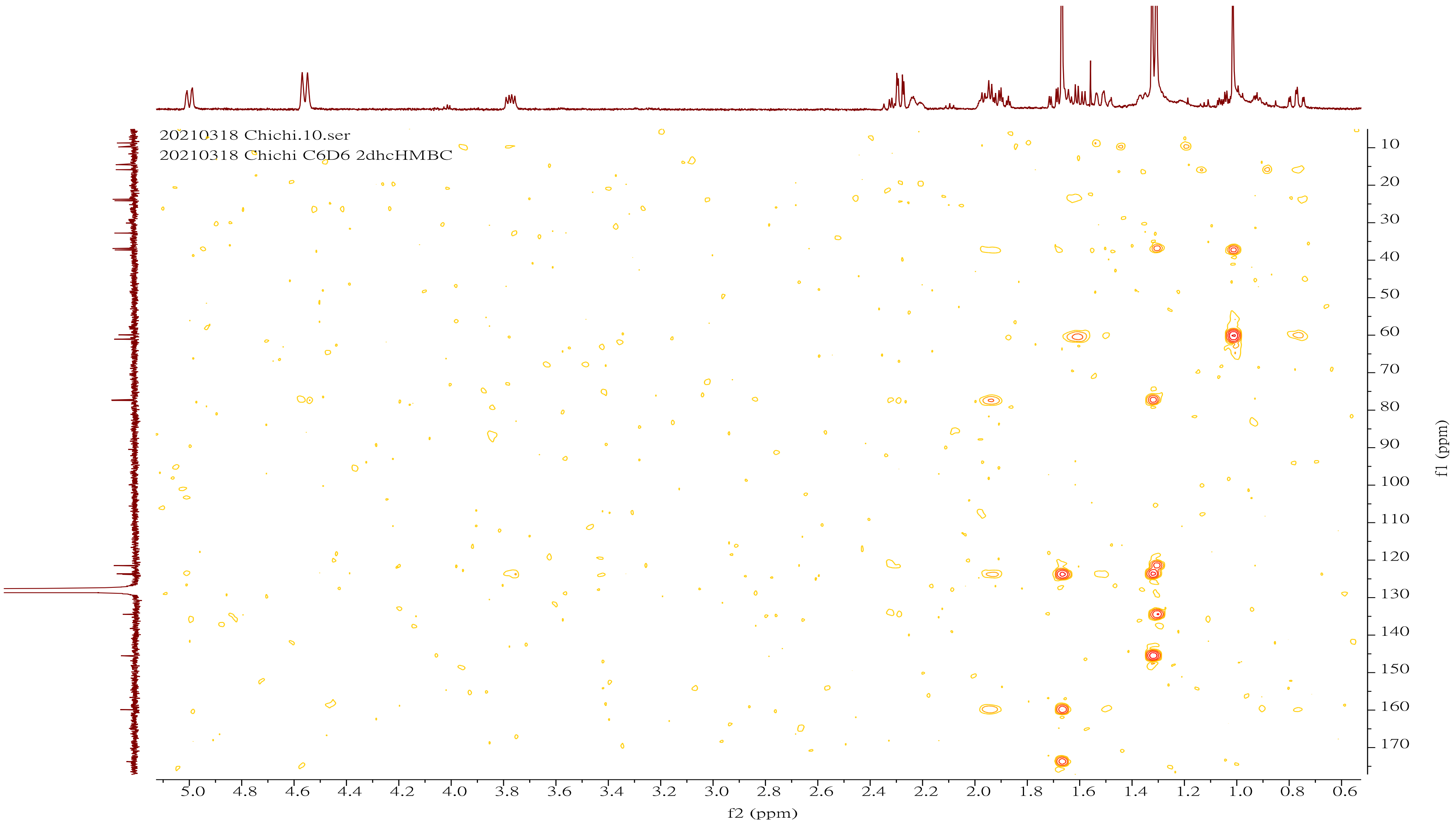

Figure S19: HMBC spectrum of **1** in C_6D_6

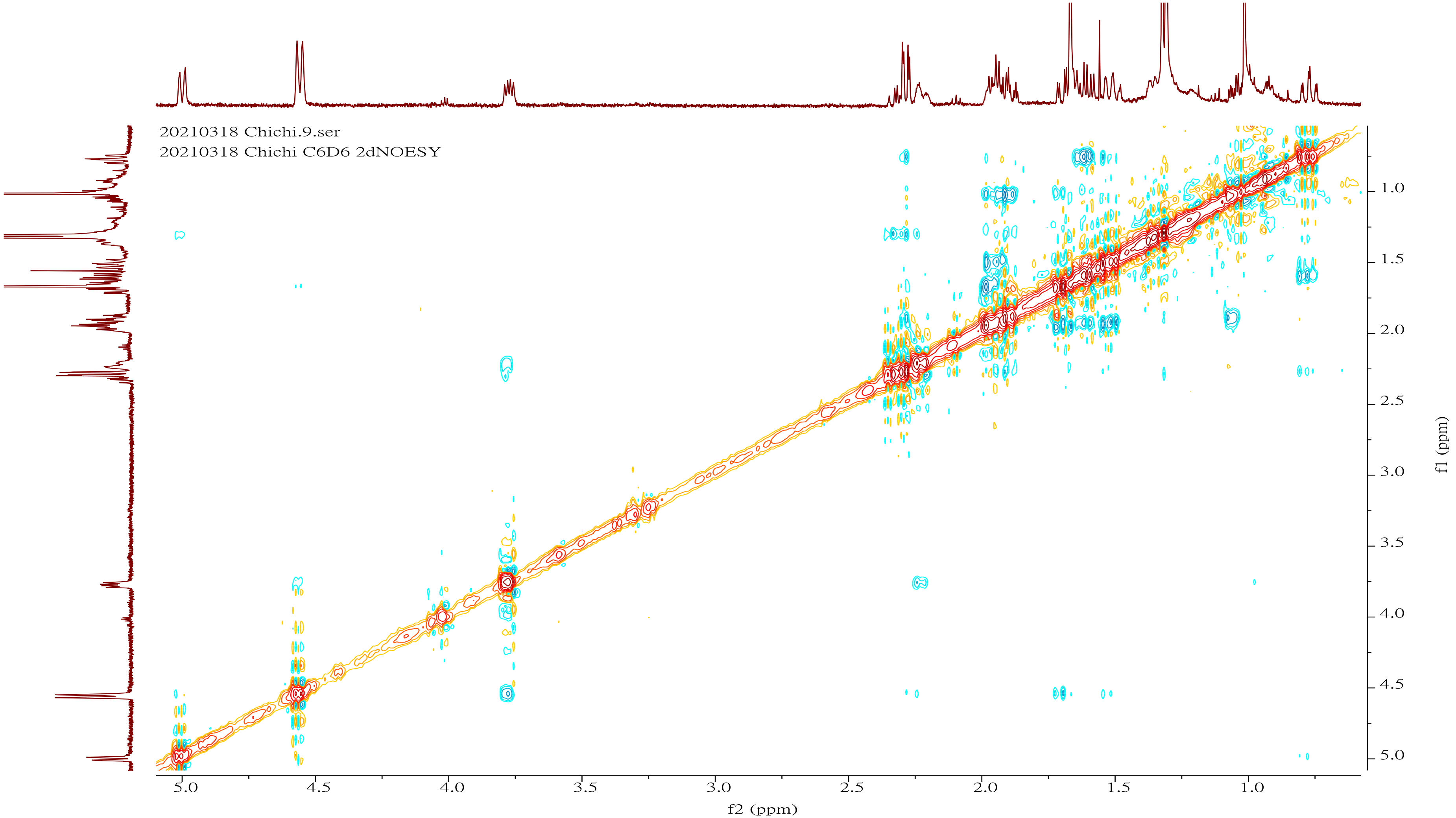

Figure S20: NOESY spectrum of **1** in C_6D_6

Spectroscopic data of cherbonolide N (**2**)

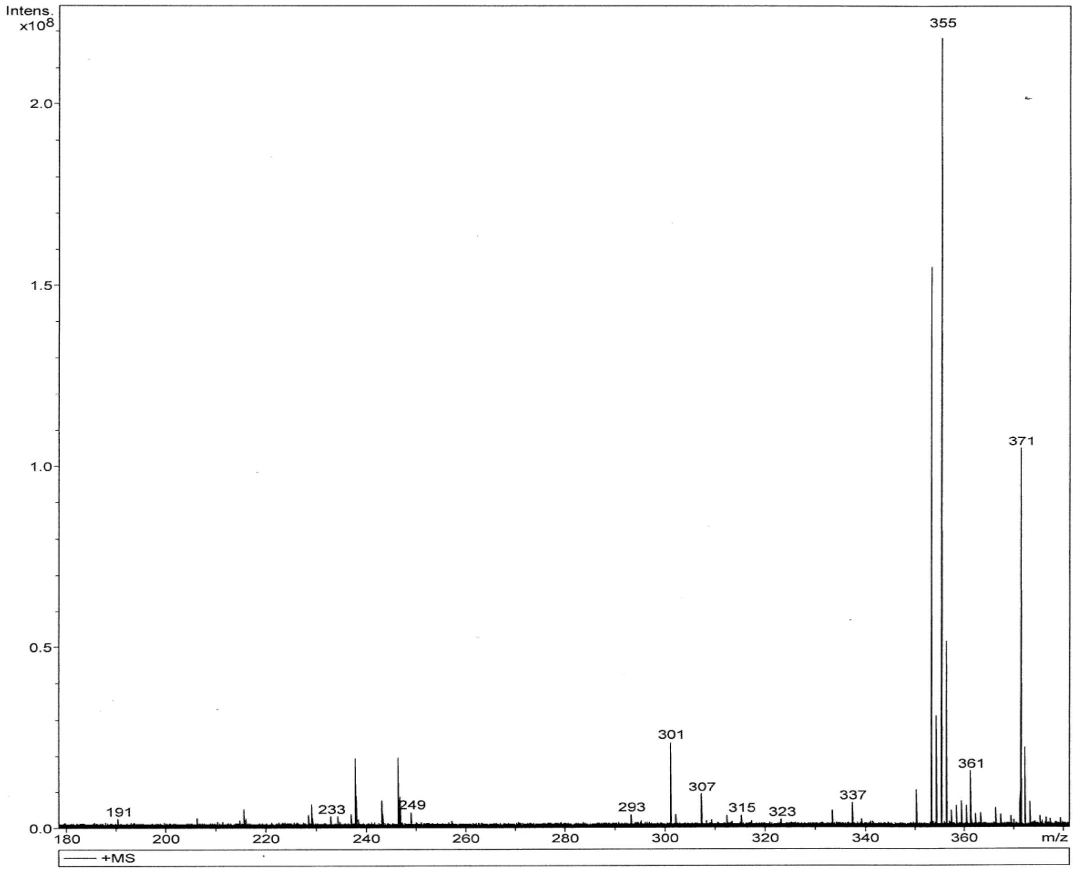

Figure S21: ESIMS spectrum of **2**

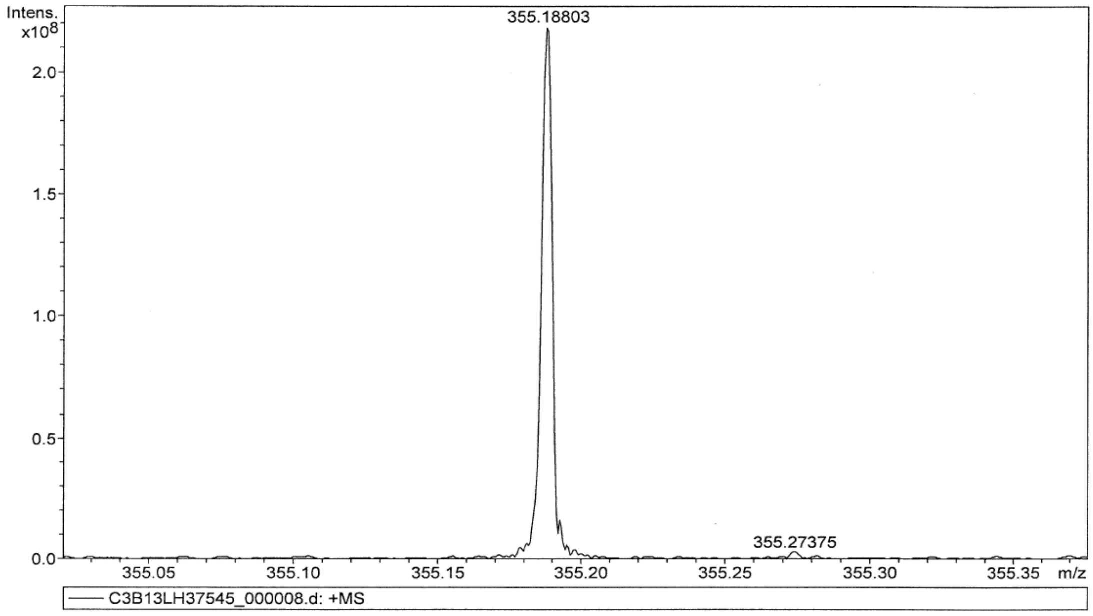

Meas. m/z	#	Formula	Score	m/z	err [mDa]	err [ppm]	mSigma	rdb	e⁻ Conf	N-Rule
355.18803	1	C 20 H 28 Na O 4	100.00	355.18798	-0.05	-0.15	12.4	6.5	even	ok

Figure S22: HRESIMS spectrum of **2**

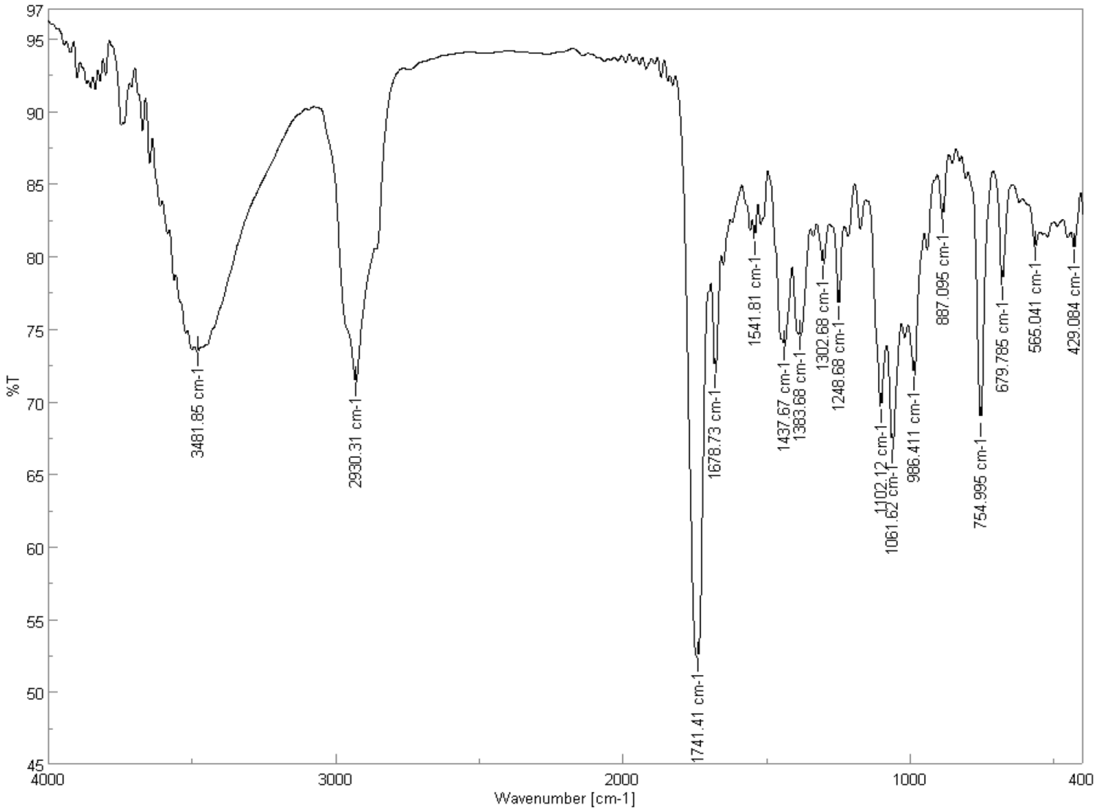

Figure S23: IR spectrum of **2**

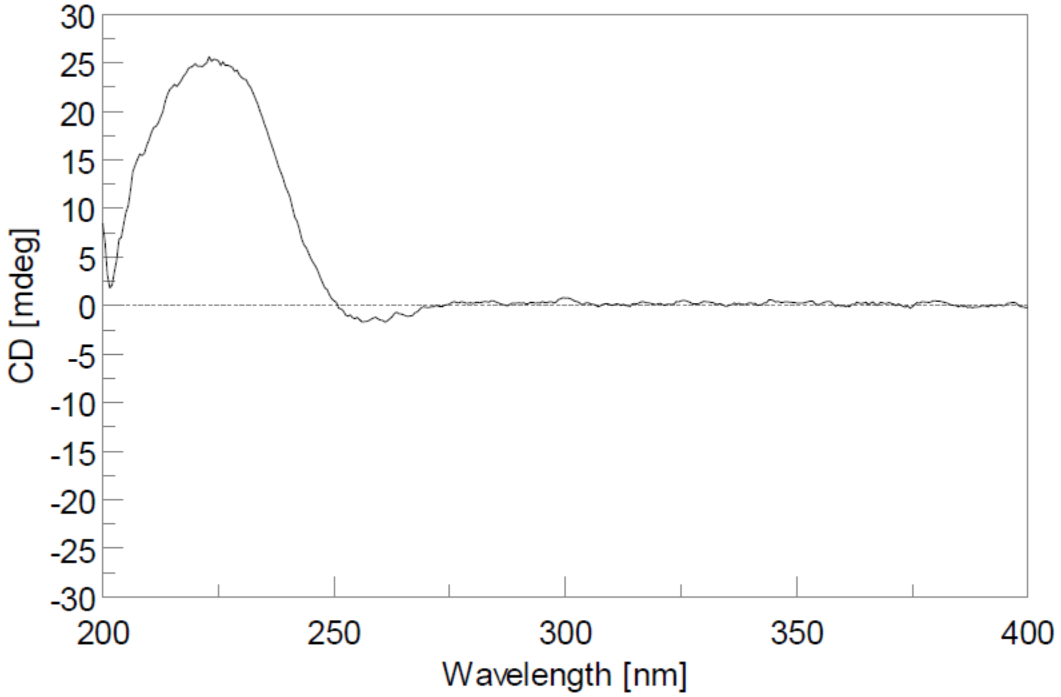

Figure S24: CD spectrum (1.2×10^{-4} M, MeOH) of **2**

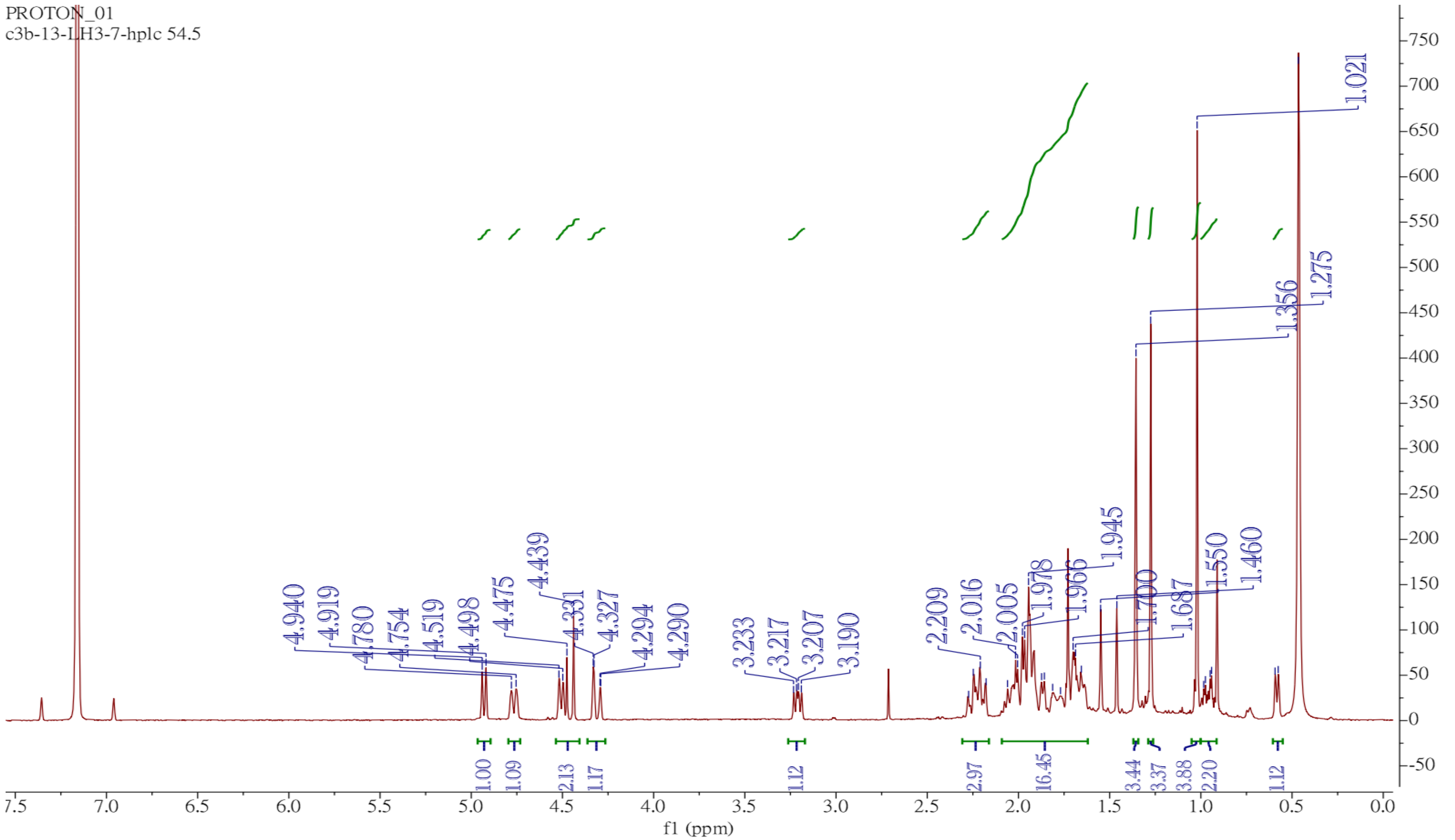

Figure S25: ^{1}H NMR spectrum of **2** in C_6D_6 at 400 MHz

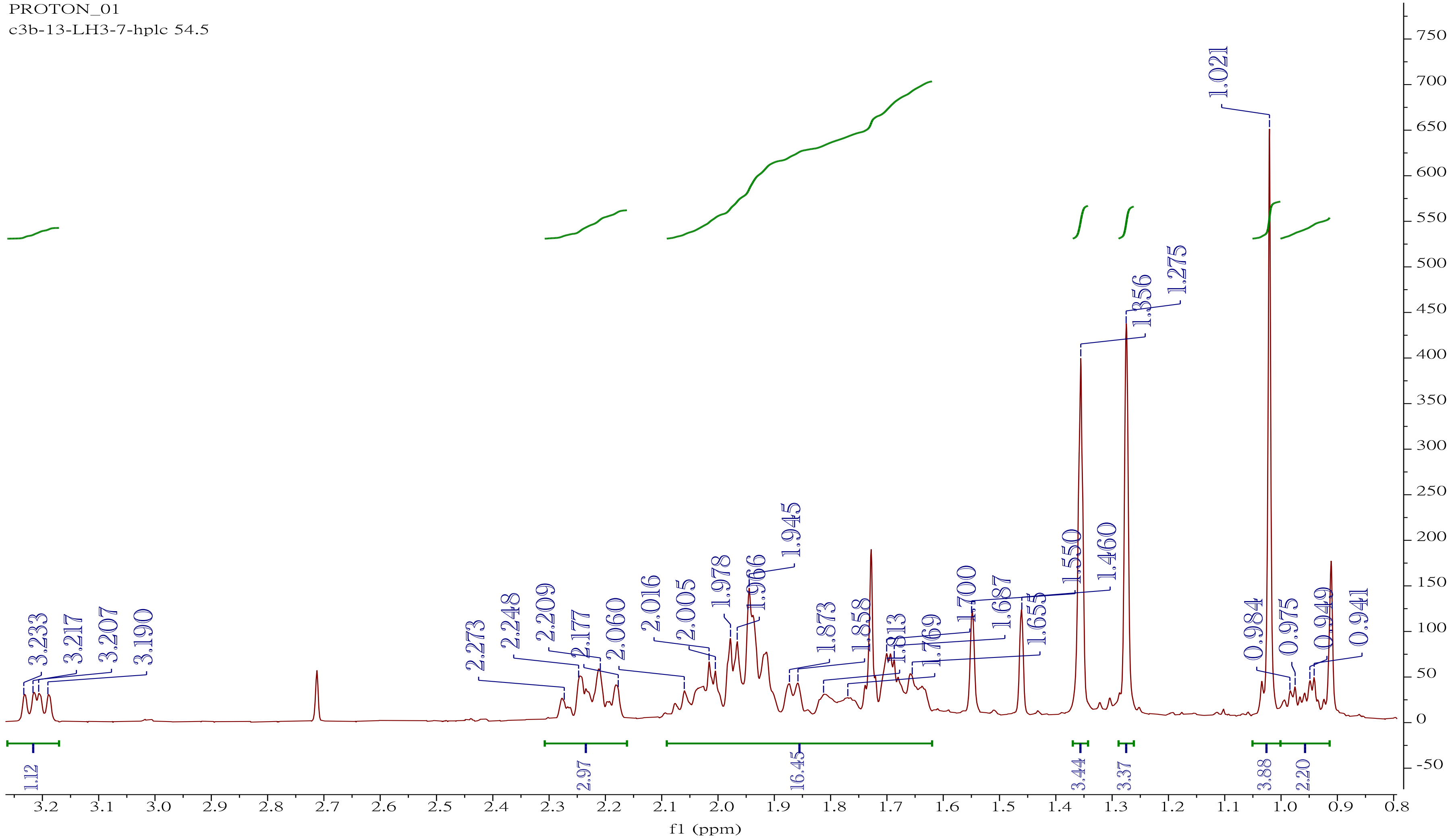

Figure S26: ^{1}H NMR spectrum (from 0.8 to 3.2 ppm) of **2** in C_6D_6 at 400 MHz

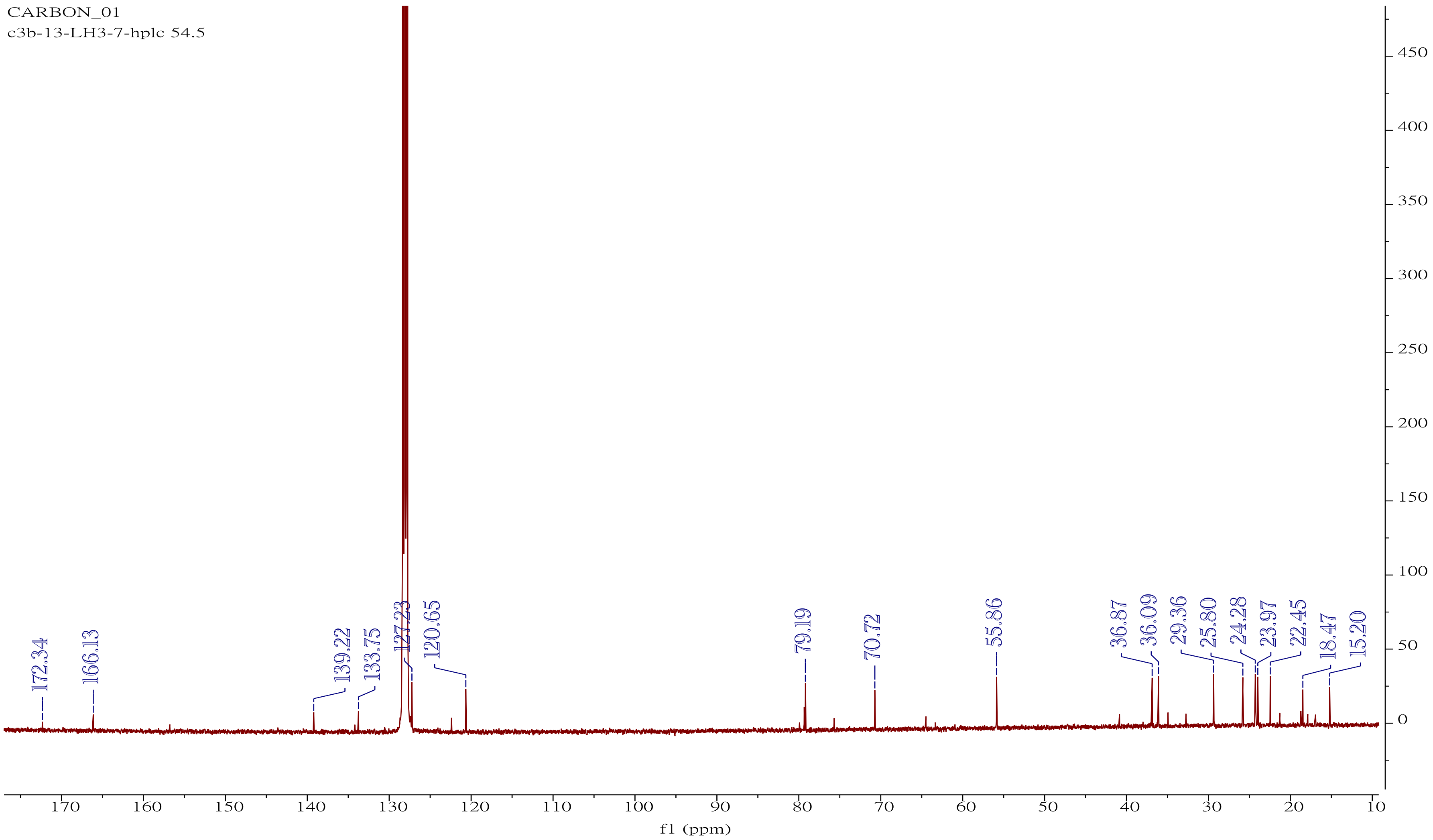

Figure S27: ^{13}C NMR spectrum of **2** in C_6D_6 at 100 MHz

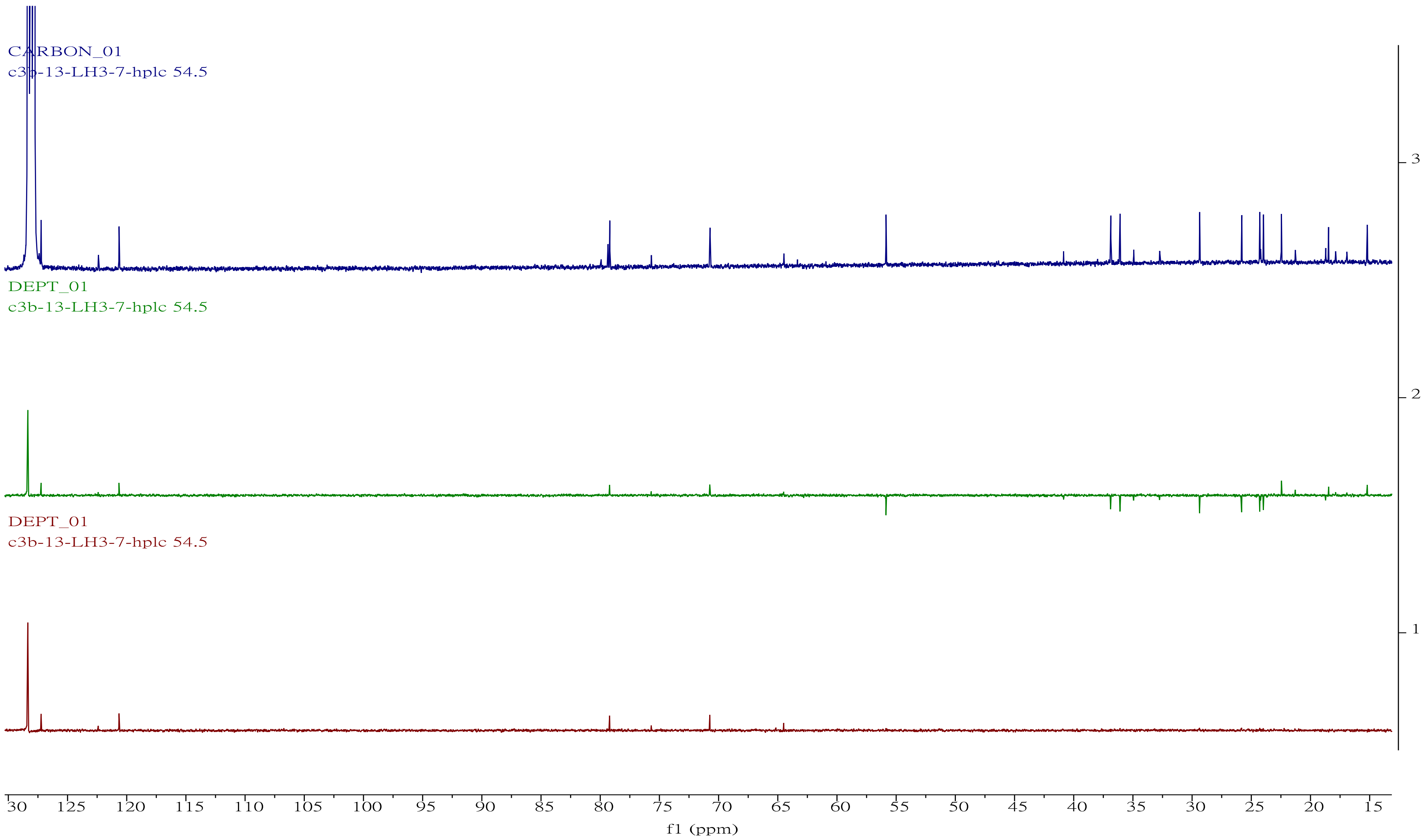

Figure S28: DEPT spectrum of **2**

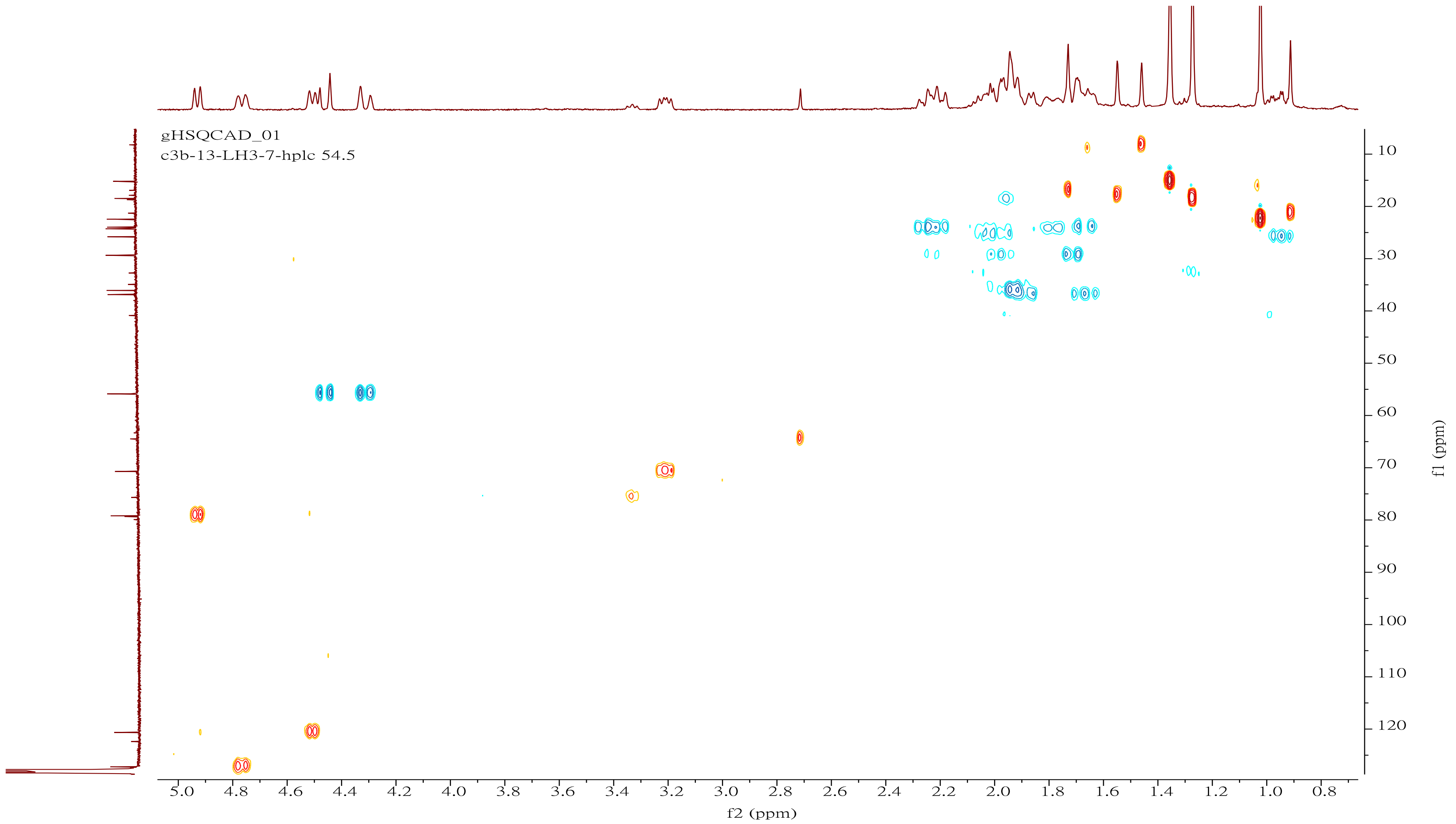

Figure S29: HSQC spectrum of **2**

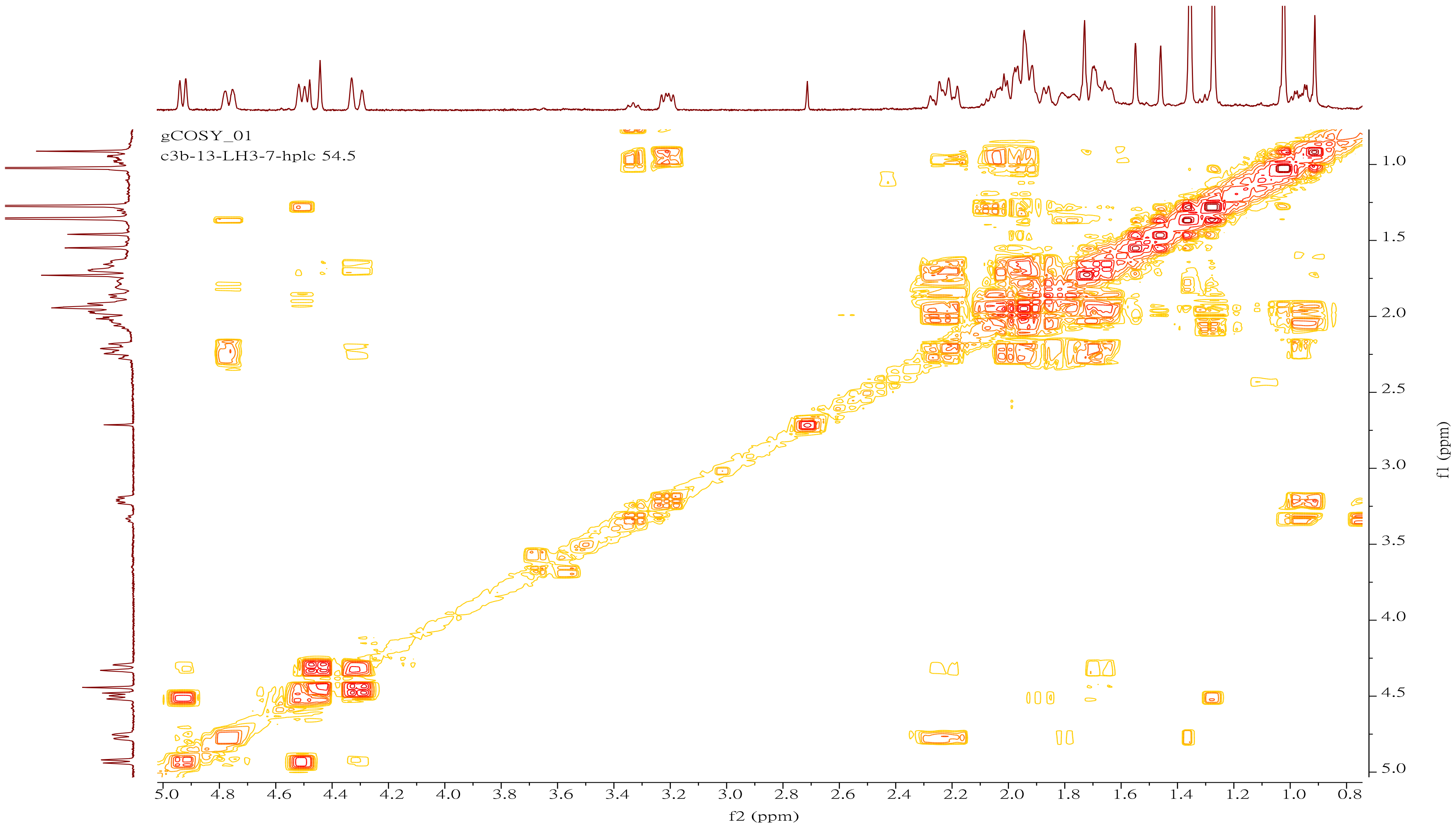

Figure S30: COSY spectrum of **2**

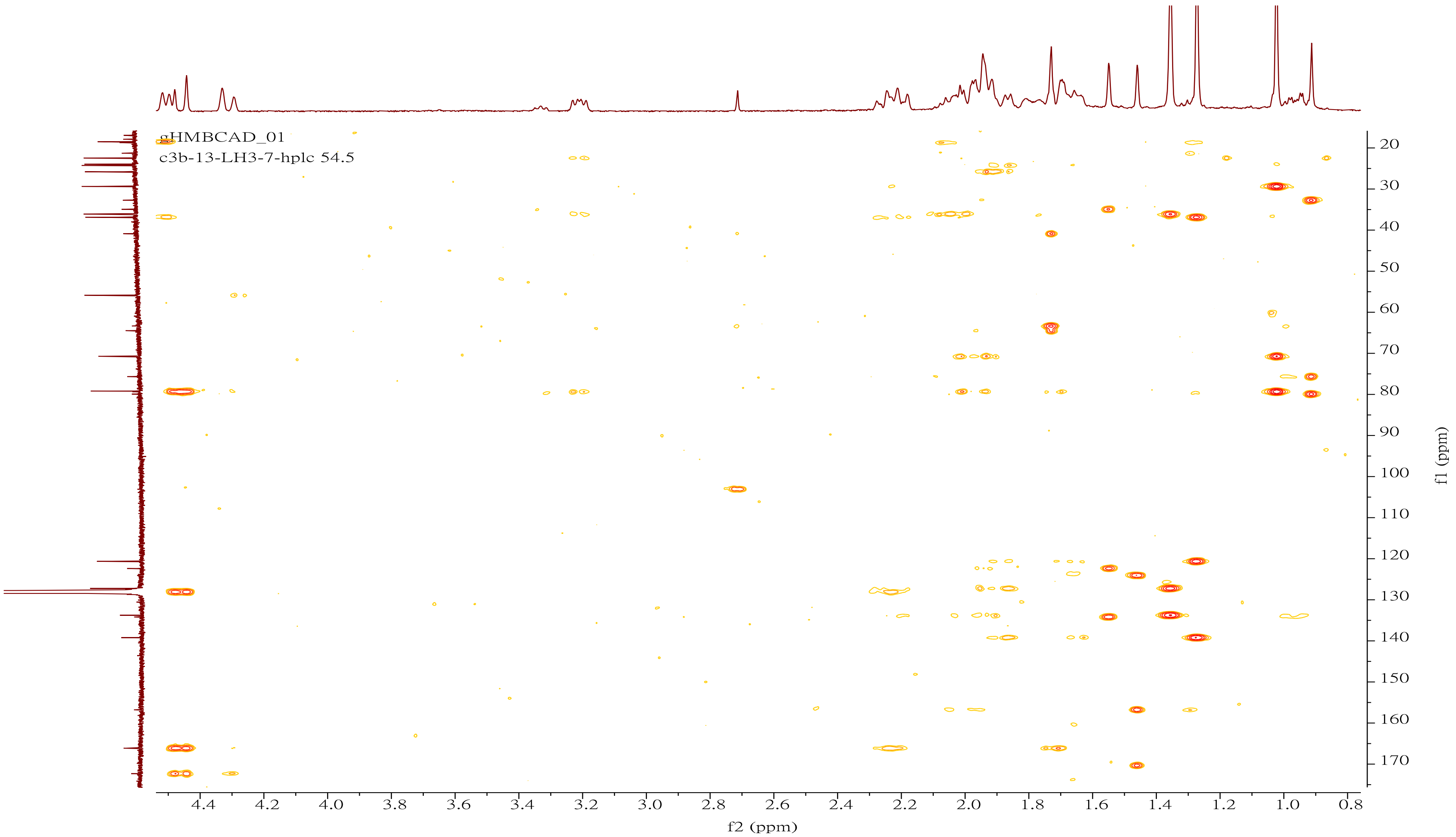

Figure S31: HMBC spectrum of **2**

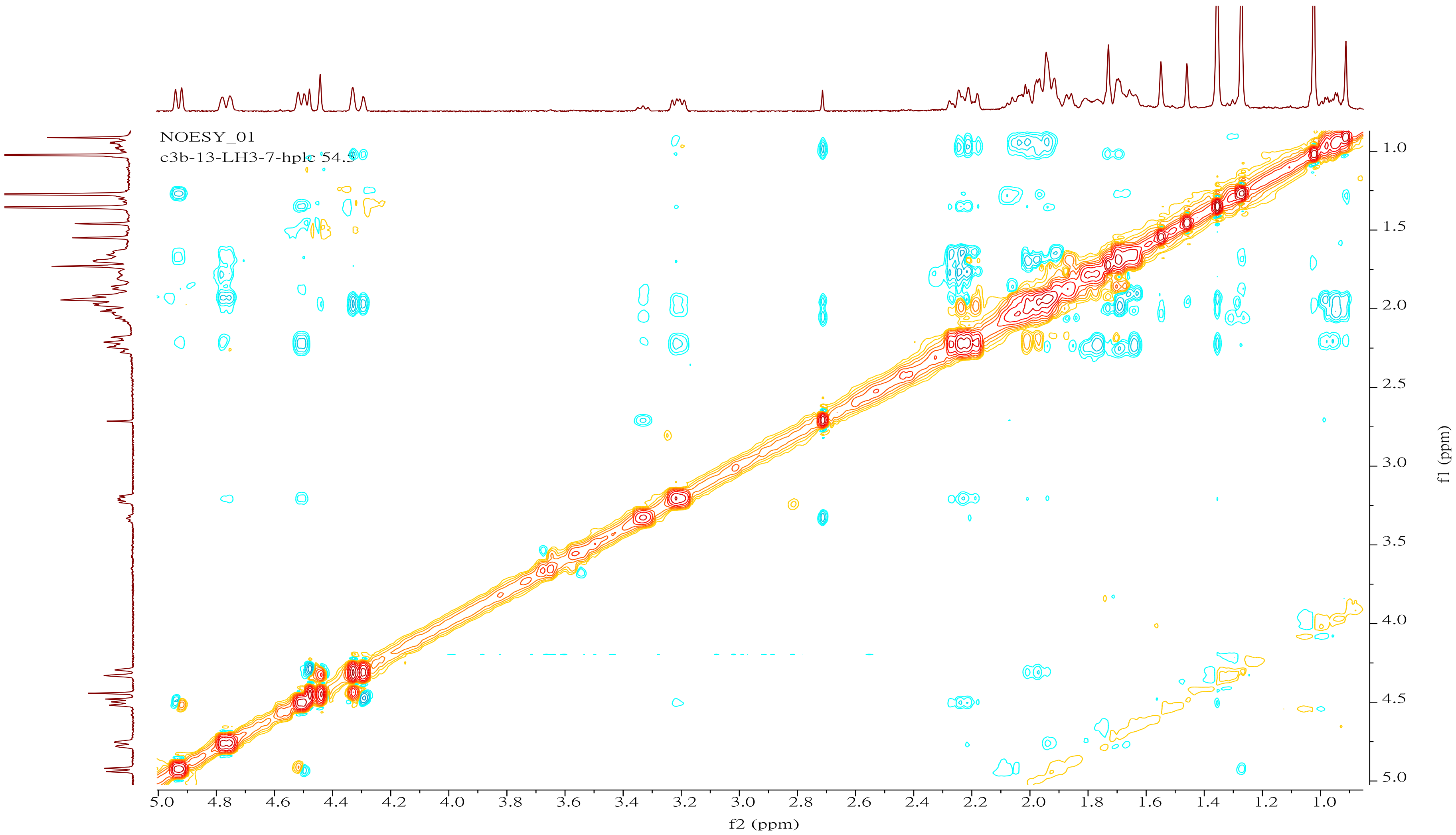

Figure S32: NOESY spectrum of **2**

Spectroscopic data of isosarcophine (*3*)

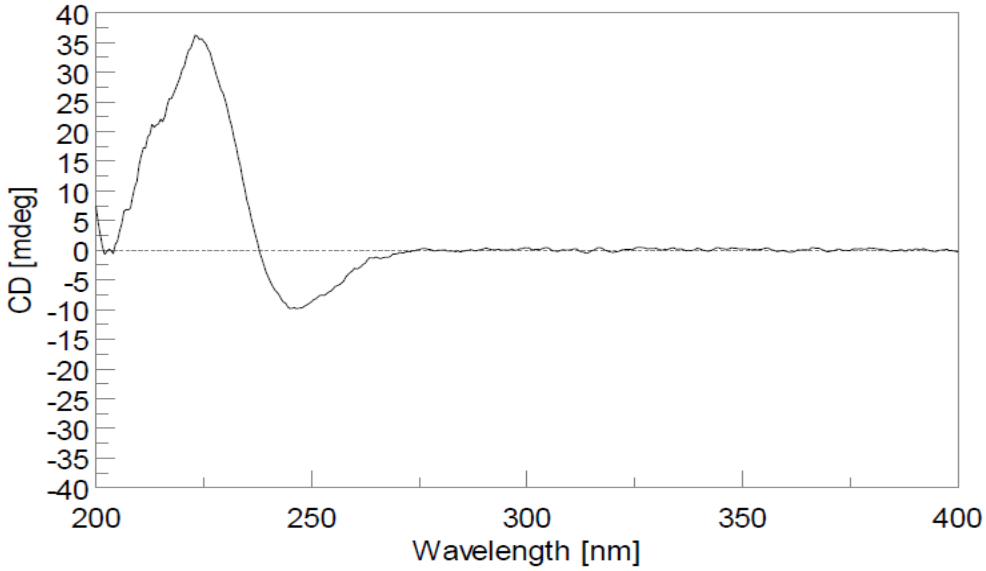

Figure S33: CD spectrum (1.6 × 10^{-4} M, MeOH) of isosarcophine (**3**)
